# *Caenorhabditis elegans* ATPase inhibitor factor 1 (IF_1_) MAI-2 preserves the mitochondrial membrane potential (Δ*ψ*_m_) and is important to induce germ cell apoptosis

**DOI:** 10.1371/journal.pone.0181984

**Published:** 2017-08-22

**Authors:** L. P. Fernández-Cárdenas, E. Villanueva-Chimal, L. S. Salinas, C. José-Nuñez, M. Tuena de Gómez Puyou, R. E. Navarro

**Affiliations:** 1 Departamento de Biología Celular y Desarrollo, Instituto de Fisiología Celular, Universidad Nacional Autónoma de México, Ciudad de México, México; 2 Departamento de Bioquímica y Biología Estructural, Instituto de Fisiología Celular, Universidad Nacional Autónoma de México, Ciudad de México, México; National Institute of Environmental Health Sciences, UNITED STATES

## Abstract

When the electrochemical proton gradient is disrupted in the mitochondria, IF_1_ (Inhibitor Factor-1) inhibits the reverse hydrolytic activity of the F_1_F_o_-ATP synthase, thereby allowing cells to conserve ATP at the expense of losing the mitochondrial membrane potential (Δ*ψ*_m_). The function of IF_1_ has been studied mainly in different cell lines, but these studies have generated contrasting results, which have not been helpful to understand the real role of this protein in a whole organism. In this work, we studied IF_1_ function in *Caenorhabditis elegans* to understand IF_1_´s role *in vivo*. *C*. *elegans* has two inhibitor proteins of the F_1_F_o_-ATPase, MAI-1 and MAI-2. To determine their protein localization in *C*. *elegans*, we generated translational reporters and found that MAI-2 is expressed ubiquitously in the mitochondria; conversely, MAI-1 was found in the cytoplasm and nuclei of certain tissues. By CRISPR/Cas9 genome editing, we generated *mai-2* mutant alleles. Here, we showed that *mai-2* mutant animals have normal progeny, embryonic development and lifespan. Contrasting with the results previously obtained in cell lines, we found no evident defects in the mitochondrial network, dimer/monomer ATP synthase ratio, ATP concentration or respiration. Our results suggest that some of the roles previously attributed to IF_1_ in cell lines could not reflect the function of this protein in a whole organism and could be attributed to specific cell lines or methods used to silence, knockout or overexpress this protein. However, we did observe that animals lacking IF_1_ had an enhanced Δ*ψ*_m_ and lower physiological germ cell apoptosis. Importantly, we found that *mai-2* mutant animals must be under stress to observe the role of IF_1_. Accordingly, we observed that *mai-2* mutant animals were more sensitive to heat shock, oxidative stress and electron transport chain blockade. Furthermore, we observed that IF_1_ is important to induce germ cell apoptosis under certain types of stress. Here, we propose that MAI-2 might play a role in apoptosis by regulating Δ*ψ*_m_. Additionally, we suggest that IF_1_ function is mainly observed under stress and that, under physiological conditions, this protein does not play an essential role.

## Introduction

F_1_F_o_-ATP synthase is an evolutionarily conserved enzyme that synthesizes ATP from ADP and P_i_ using a H^+^ gradient that the electron transport chain complexes generate [1,2]. When the electrochemical proton gradient that spans the mitochondrial inner membrane collapses—for example, during ischemia—ATP synthase catalyzes the back reaction and hydrolyzes ATP (F_1_F_o_-ATPase) to conserve the mitochondrial membrane potential (Δ*ψ*_m_) [3–5]. Therefore, ATPase Inhibitor Factor 1 (IF_1_) regulates F_1_F_o_-ATPase hydrolyzing activity and maintains cellular ATP [3,6,7]. Several groups have attributed different roles to IF_1_ apart from its canonical function in regulating F_1_F_o_-ATPase; however, some of them are contradictory. For example, some studies have proposed that IF_1_ regulates the mitochondria volume through autophagy [8] and promotes the dimeric form of F_1_F_o_-ATP synthase complexes [4,9,10] that may influence mitochondria cristae formation [4]. Furthermore, Faccenda *et al*. observed that the disruption of mitochondria cristae, due to the down-regulation of IF_1_, increased cytochrome *c* liberation and apoptosis [11]. By contrast, Fujikawa *et al*. (2012) from the Yoshida group found that IF_1_ knockdown (KD) HeLa cells were neither affected in mitochondria volume nor in diminished cristae formation [12]; the same observation was made by other groups [13–15]. Fujikawa *et al*. explained that these differences might be due to the methods used for silencing IF_1_ expression [12]. While Campanella and Faccenda [4,11] used transient knockdown, Fujikawa *et al*. suppressed IF_1_ permanently [12]. Recently, the Campanella and Yoshida groups performed a more extensive analysis of mitochondria in the permanent KD cells and observed that, indeed, mitochondria cristae and volume are affected when IF_1_ is silenced [16].

It has also been observed that IF_1_ protects cultivated cells from stress. Campanella *et al*. (2008) found that IF_1_ down-regulated cells showed increased cell death when cultivated in glucose-free medium and exposed to anoxia [4]. Similarly, Fujikawa *et al*. (2012) exposed IF_1_-KD cells to 2-deoxyglucose (glycolysis inhibitor) and cyanide (respiratory inhibitor) and observed that these cells died faster than control cells and were more sensitive to paraquat [12]. By contrast, Chen *et al*. found that the loss of IF_1_ increased cell survival against complex III inhibition, proposing that IF_1_ inhibition might help ameliorate severe mitochondrial respiratory chain disorders [14]. To explain IF_1_-KD susceptibility to stressful conditions, Campanella *et al*. and Fujikawa *et al*. [4,12] suggested that IF_1_-KD cells continue depleting ATP, and the lack of ATP makes cells more sensitive to stress. Additionally, Chen *et al*. [14] proposed that IF_1_ knockout (KO) boosts Δ*ψ*_m_ with the loss of ATP, improving mitochondria health and promoting cell survival.

One way to conciliate these results is to study IF_1_ function in model organisms. In zebrafish, *atpif1a* loss-of-function mutants and morpholino KD fish lack hemoglobinized cells, resulting in anemia [13]. The mechanism that leads to the anemic phenotype was elucidated by silencing *atpif1* in mouse erythroleukemia cells (MELs). *atpif1* KD cells showed an increase in Δ*ψ*_m_, leading to an alkaline pH in the mitochondria and altering the activity of the mitochondrial FECH enzyme, which synthesizes the heme group [13]. Surprisingly, IF_1_-KO mice showed no evident phenotype and had no differences compared with wild-type animals in ATP synthesis or hydrolysis, F_1_F_o_-ATPase dimer content or autophagy [17]. Recently, a more extensive analysis of the mitochondria ultra-structure in the KO mice showed fewer cristae formation and scarcer mitochondria per field [16].

To study additional IF_1_ function *in vivo*, we used the nematode *Caenorhabditis elegans*. The *C*. *elegans* genome encodes two ATPase inhibitor proteins MAI-1 and MAI-2 [18]. Previously, Ichikawa *et al*. (2006) characterized *C*. *elegans* MAI-1 and MAI-2 proteins localization and biochemical parameters in yeast and found that both proteins had inhibitory activity for the ATPase. However, MAI-1 is localized in the yeast´s cytoplasm, while MAI-2 is localized in the yeast´s mitochondria [19]. To corroborate whether MAI-1 and MAI-2 localization in yeast was conserved in *C*. *elegans*, we generated GFP::MAI-1 and MAI-2::GFP transgenic animals and observed that MAI-2::GFP is localized in the mitochondria and is expressed ubiquitously, whereas GFP::MAI-1 is expressed in specific tissues of the nematode in a non-mitochondrial pattern. Our work confirms that MAI-1 is not a mitochondrial protein and, because MAI-2 has a more conventional expression pattern and role for IF-1, we studied *mai-2* function.

We generated, by CRISPR/Cas9 genome editing, two different mutant alleles for *mai-2*. In contrast to previous phenotypes described in KD cell lines, we found that *mai-2* mutants displayed no significant differences in the oxygen consumption rate, total ATP concentration, mitochondrial network and F_1_F_o_-ATP synthase dimerization. Interestingly, we found that *mai-2* mutant animals had hyperpolarized mitochondria and showed decreased physiological germ cell apoptosis. When the mutants were exposed to different stresses, we found that the germ cell apoptosis response was decreased compared with that of the control, suggesting that MAI-2 affects the germ cell response to stress. Furthermore, when we exposed *mai-2* mutants to heat shock, oxidative stress and mitochondria-disturbing agents such as cyanide, an electron transport chain inhibitor and a CCCP uncoupler, we observed that *mai-2* mutant animals were more vulnerable than wild-type. Our results showed that, in an organism such as *C*. *elegans*, MAI-2 appears to be non-essential under physiological conditions; however, when animals encounter stress, the survival of the whole organism seem to be affected.

## Materials and methods

### Strains

*C*. *elegans* strains were maintained on NGM-lite agar plates seeded with *Escherichia coli* (*E*. *coli*) strain OP50 bacteria [20,21]. Strains are indicated in Table 1.

**Table 1:** Strains. List of strains that were used in this study, their genotype, and optimal temperatures is shown.

Strains	Genotype	Growth Tempertaure	References
RN69	[*Pmai-2*::*mai-2*::*GFP*::*mai-2 3’*UTR*; unc-119*(+)]*	24°C	This work
RN80	[*Pmai-2*::*mai-2*::*GFP*::*mai-2 3’UTR; unc-119*(+)]*;xmSi01*	24°C	This work
EG6699	*ttTi5605 II; unc-119(ed3) III oxEx1578*	15°C	[26]
RN37	*xmSi31*[*Pmai-1*::*mai-1*::*mCherry*::*mai-1 3’*UTR, *Cbr unc-119*(+)]II	24°C	This work
RN38	*xmSi32*[*Pmai-1*::*GFP*::*mai-1*::*mai-1 3’*UTR, *Cbr unc-119*(+)]II	24°C	This work
RN15	*xmSi01*[*Pmex-5*::*tomm-20*::*mcherry*::*tbb-2 3’*UTR*; Cbr unc-119*(+)]II	24°C	This work
MD701	*bcIs39*[*Plim-7*::*ced-1*::*GFP; lin-15*(*+*)]V	24°C	[51]
RN70	*mai-2(xm18)* IV*; bcIs39* V	24°C	This work
RN71	*mai-2(xm19)* IV*; bcIs39* V	24°C	This work
SD1347	*ccIs4251*[*Pmyo-3*::*GFP*::*LacZ+Pmyo-3*::*mitoGFP+dpy-20*(*+*)]I	20°C	[52]
RN79	*mai-2(xm18) IV; ccIs4251* I	20°C	This work

* The LG for the insertion has not been defined.

### Generation of translational reporters

**Fig 1:**
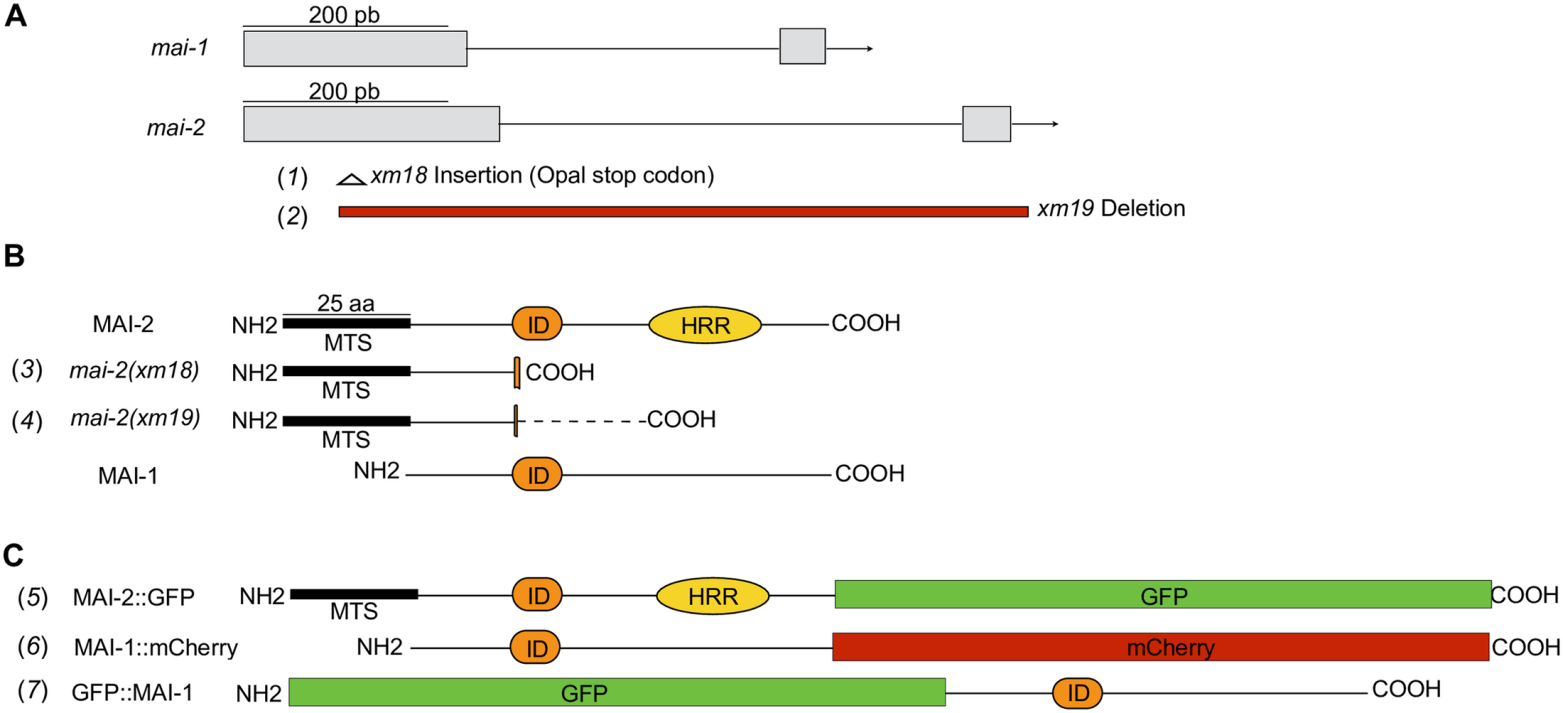
Alleles and translational reporters used to study the function of mitochondria ATPase inhibitors in *C*. *elegans*. (A) The *mai-1* and *mai-2* genes are composed of 2 exons (gray rectangles) and one intron (thin line). (B) We generated two mutant alleles, *mai-2(xm18)* and *mai-2(xm19)*, by CRISPR/Cas9 genome editing. Both mutant alleles encode two truncated MAI-2 proteins that conserve the mitochondrial targeting sequence (MTS) but lack the inhibitory domain (ID) and histidine-rich region (HRR), which are essential for MAI-2 function and regulation (3,4). The dotted line (—) in the *mai-2(xm19)* product represents a randomly formed sequence of amino acids (4). (C) To study protein localization, we generated translational reporters for MAI-1 and MAI-2 (5–7). We inserted, in the carboxyl-terminal, a GFP construct for MAI-2 (5); for MAI-1, we inserted a carboxy-terminal mCherry (6) and an amino-terminal GFP (7).

The cloning of *mai-1* and *mai-2* were done using MultiSite Gateway (Thermo Fisher Scientific) and all inserts were amplified from genomic DNA. For the transgene carrying *Pmai-2*::*mai-2*::*gfp*::*mai-2* 3’*-*UTR (Fig 1C, line 5), we amplified a 1796 bp fragment upstream of *mai-2*, for the coding region a 852 bp fragment from the ATG start codon until the end of the gene without a stop codon, a 910 bp fragment of *gfp* from template pCM1.53 (Addgene #17250) was fused with a 182 bp *mai-2* 3’*-*UTR by PCR overlap extension [22]. The PCR products were gel-purified, *mai-2* promoter was cloned in the donor vector pDONRP4-P1R (Gateway vector), *mai-2* coding region was cloned in the donor vector pDONR221 (Gateway vector) and the chimera *gfp*-*mai-2* 3’-UTR was cloned in pDONRP2R-P3 (Gateway vector). The construct *Pmai-2*::*mai-2*::*gfp*::*mai-2* 3´*-*UTR was cloned into the pCFJ150 vector and were bombarded into HT1593 *unc-119(ed3)* animals [23] using standard procedures [24,25]. To study the expression pattern, we chose to study a transgenic line that showed high expression in both tissues. For *Pmai-2*::*mai-2*::*gfp*::*mai-2* 3´*-*UTR we obtained twelve independent transgenic lines, three of which showed expression in the soma and germline.

For the transgene carrying *Pmai-1*::*gfp*::*mai-1*::*mai-1* 3’-UTR (Fig 1C, line 7) we amplified a 2002 bp fragment from the intergenic region upstream *mai-1* and for the coding region containing the 3’-UTR we amplified a 932 bp fragment from the ATG start codon until the end of the longest 3’-UTR reported (Wormbase release WS224). The PCR products were gel-purified, and the promoter region was cloned by recombination into the pDONRP4-P1R, while the coding region was cloned into pDONRP2R-P3. For the *gfp* sequence we used the donor vector pCM1.53. For the transgene carrying *Pmai-1*::*mai-1*::*mCherry*::*mai-1* 3’-UTR (Fig 1C, line 6) we used the same *mai-1* promoter described above, for *mai-1* coding region we amplified a 567 bp fragment from the ATG start codon until the end of the gene without stop codon. A *mCherry* fragment of 864 bp was amplified using as template pCFJ104 (Addgene #19328) and for *mai-1* 3’-UTR we amplified a fragment of 356 bp. We fused *mCherry* fragment with *mai-1* 3’-UTR by PCR overlap extension. The PCR products were purified, and the promoter was cloned through recombination in the donor vector pDONRP4-P1R, *mai-1* coding region was cloned in donor vector pDONR221, while *mCherry*::*mai-1* 3’-UTR chimera was cloned in pDONRP2R-P3. Inserts were sequenced after cloned in the donor vectors, and the desired fragments in each donor vector were cloned in tandem into the pCFJ150 (Addgene #19329)[26].

For the generation of *Pmex-5*::*tomm-20*::*tbb-2* 3’-UTR, we amplified a fragment of 225 bp of *tomm-20* gene, *mCherry* sequence was amplified from the vector pGH8 (Addgene #19359). The PCR products were gel-purified and fused by PCR overlap extension. The fused PCR product was cloned into the pDONR221. Finally, the donor vector pCFJ183-*Pmex-5* (kindly donated by Frøkjaer-Jensen), pDONR221-*tomm-20*::*mcherry* and pCm1.36 (Addgene #17249) were mixed and cloned in tandem through recombination into the destination vector pCFJ150 vector. Primer sequences are listed in S1 Table.

For *Pmai-1*::*gfp*::*mai-1*::*mai-1* 3*’-*UTR, *Pmai-1*::*mai-1*::*mCherry*::*mai-1 3’*UTR and *Pmex-5*::*tomm-20*::*mCherry*::*tbb-2* 3’-UTR transgenes were inserted by single copy insertion methodology [26,27]. DNA mixtures were microinjected into the strain EG6699 and selection of the transgenic animals was done a previously reported with modified microinjection mixtures [25]. The microinjection mixtures used were: target vector pCFJ150 with *Pmai-1*::*gfp*::*mai-1*::*mai-1* 3’-UTR (30 ng/ μl), pMA122 (10 ng/μl) (Addgene #34873), pCFJ601 (50 ng/μl) (Addgene #34874), pCFJ104 (5 ng/μl), pGH8 (Addgene #19359) and pCFJ90 (2 ng/μl) (Addgene #19327); pCFJ150 with *Pmai-1*::*mai-1*::*mCherry*::*mai-1* 3’-UTR transgene (30 ng/μl), pMA122 (10 ng/μl), pCFJ601 (50 ng/μl), pCFJ421 (2.5 ng/μl) (Addgene #34876), pCFJ420 (5 ng/μl) (Addgene #34877), pCFJ66 (10 ng/μl) (Addgene #24981); pCFJ150 with *Pmex-5*::*tomm-20*::*mcherry*::*tbb-2* 3’*-*UTR (10 ng/μl), pCFJ601 (10 ng/μl), pCFJ104 (10 ng/μl), pGH8 (10 ng/μl), pCFJ90 (2 ng/μl).

For *Pmai-1*::*gfp*::*mai-1*::*mai-1* 3*’-*UTR we obtained one transgenic line named *xmSi32*[*Pmai-1*::*gfp*::*mai-1-3’-*UTR*; Cbr-unc-119* (+)]II and for *Pmai-1*::*mai-1*::*mCherry*::*mai-1 3’*UTR we obtained two independent lines with the same expression pattern, and we chose to work with *xmSi31*[*Pmai-1*::*mai-1*::*mCherry*::*mai-1-3’-*UTR; *Cbr-unc-119* (+)]II.

### Image acquisition and processing of translational reporters

The embryos expressing MAI-2::GFP were observed by fluorescence microscopy to detect GFP expression using a Nikon Eclipse E600 microscope equipped with an AxioCam MRc camera (Zeiss). The pictures were captured using AxioVision software (Zeiss) and deconvolved using ImageJ software [28] with Parallel Spectral Deconvolution and Diffraction PSF 3D plugins. Larvae and adult animals expressing MAI-2::GFP and TOMM-20::mCherry, GFP::MAI-1 and MAI-1::mCherry were observed by confocal imaging using an Olympus Fluoview Confocal microscope FV10i (Olympus) and images were processed with ImageJ software.

### Generation of *mai-2* alleles

CRISPR/Cas9 genome editing was used to generate *mai-2(xm18)* and *mai-2(xm19)* alleles [29,30]. We targeted a site localized in the first exon of *mai-2* with a single guide (sgRNA) to be cut by Cas9 with the sequence GGATCGATCCGCGACGCCGG. The protospacer-associated motif (PAM) is at position 3,386,025 (Wormbase release WS224). To generate the *mai-2* sgRNA we replaced by PCR the *unc-119* in the pU6::*unc-119* sgRNA vector (Addgene #46169) with the desired *mai-2* sequence using primers listed in S1 Table. The PCR product was digested with DpnI, purified pU6::*mai-2* sgRNA was confirmed by sequencing. Single-strand oligonucleotides (ssDNA) were used as repair templates [30]. *mai-2* ssDNA1 repair template consisted of a 5’ upstream homology arm of 83 nt before the modification site which consisted in the insertion of a PvuII restriction enzyme site that introduced an opal stop codon, and a 78 nt 3’ downstream homology arm. To generate a deletion of *mai-2* gene we used ssDNA2 that consisted of a 67 nt 5´upstream homology arm and a 71 nt downstream homology arm of *mai-2* 3’-UTR. We microinjected the following vectors: *Peft-3*::*cas-9-SV40-NLS*::*tbb-2* 3’-UTR (50 ng/μl) (Addgene #46168), pU6::*mai-2* sgRNA (45 ng/μl), pCFJ104 (5 ng/μl) and repair templates ssDNA 1 or 2 (30 ng/μl) in 30–50 young adults of N2 strain. F_1_ with mCherry expression were picked individually to plates and allowed to lay eggs for one day, then F1 were transfered into lysis buffer and were screened by PCR followed in the case of allele *mai-2(xm18)* by restriction enzyme digestion to detect insertion. Primer sequences are listed in S1 Table.

### RT-PCR

The RNA purification and cDNA synthesis was done as previously reported [25]. The total RNA was isolated from approximately 7,000 one-day-old adult animal hermaphrodites using TRIzol (Life Technologies) and purified via chloroform and isopropanol precipitation. 0.5–1 μg of RNA was reverse transcribed into cDNA using Im-Prom II reverse transcriptase (Promega) and oligo(dT) primers. Semi-quantitative reverse transcriptase-PCR was performed with primers designed specifically for *mai-2* (listed in S1 Table) and normalized with *act-4*. Densitometric analysis was done using Fiji (ImageJ) software.

### Lifespan, fertility and embryonic lethality

Lifespan was done as previously described [31]. An experimental pool of synchronized 50 animals from N2 and *mai-2(xm18)* strains were used, and lost or animals that died prematurely were replaced from a back-up pool. Lifespan measurements were done al 20°C.

The fertility assay and embryonic lethality were done as previously described [32]. N2 and *mai-2* mutants were grown at 20°C and were individually selected as L4 larvae and then transferred to new plate every 24 h over the course of 4 days. Embryos that did not hatch within 24 h after being laid were considered dead.

### Mitochondria imaging

MitoTracker Red CMX ROS (#M7512; Molecular Probes) was diluted in DMSO (1 mM stock solution). Before staining, stocks were diluted in M9 buffer at 5 μM and one-day-old adult animals were incubated for 20 min at 20°C [33]. Animals were immobilized with tetramisol 10 mM and mounted on 2% agarose pads for microscopic examination. For TMRM (#T-668; Molecular Probes) we modified procedure from previous method [34,35]. We diluted TMRM in DMSO (0.1 M stock solution) and added to NGM-lite agar medium at a final concentration of 30 μM, we left the plates to dry overnight and seeded *E*. *coli* OP50 for 24 h in the dark. For embryo imaging we transferred approximately 30 one-day-old adults of each strain to the TMRM plates, incubated at 20°C for 15 h, and dissected animals for embryo extraction. For complete animal staining we incubated L4 animals in TMRM plates for 15 h. Stained embryos and animals were mounted on 2% agar pads for microscopy. Images were acquired under the same exposure and to calculate the level of fluorescence its integrated density was measured in ImageJ software by selecting the whole area of P_1_ cell (embryos) and the whole animal. The corrected total fluorescence was calculated as CTCF = (integrated density-(selected area x mean fluorescence of background readings). For visualizing the mitochondria network in the body wall muscle cells we used synchronized L4 larvae from the transgenic strain SD1347 crossed with *mai-2(xm18)*. Animals were anesthetized with tetramisol diluted in M9 (10 mM), and mounted on an agarose pad (2%). A cover slide was put on top of the agarose pads and to rotate worms for muscle cells visualization, the cover slide was push carefully. We quantified all mitochondria per muscle cell as tubular (wild-type), fragmented, elongated/highly connected or aggregated mitochondria. Mitochondria visualization we performed using a Nikon Eclipse E600 microscope equipped with an AxioCam MRc camera (Zeiss).

### Oxygen consumption

Oxygen consumption rates were measured as previously described [36], with modifications using a Strathkelvin Instruments 782 Oxygen Meter (North Lanarkshire, Scotland, UK) interfaced to a computer. Approximately, 10,000 animals/ml in L4 to young adults were collected, washed 3 times in M9 and incubated for 30 min in constant agitation to empty the digestive system. 15 μl of slurry pellet of worms were delivered into a water-jacketed chamber Mitocell (MT200) at 20°C with 100 μl of M9 on it. Oxygen consumption was recorded until linear and stopped with NaCN to abolish oxygen consumption. Worms were recovered after respiration measurements and collected for protein quantification by Lowry assay [37,38]. Rates were normalized to protein content.

### ATP quantification

ATP quantification was done as previously described with slight modifications [35]. 50 one-day-old adult hermaphrodites were collected in 50 μl of M9 buffer and frozen in liquid N_2_. Frozen worms were immersed in boiling water for 15 min, cooled and centrifuged at 2000 rpm for 5 min to pellet insoluble debris. The supernatant was moved to a fresh tube and diluted 5-fold before measurement. ATP content was determined using the Roche ATP bioluminescent assay kit HS II (Roche Applied Science) and a POLARstar Omega luminometer (BMG LABTECH). ATP levels were normalized to total protein content.

### Mitochondria isolation, blue native electrophoresis and SDS-PAGE

About 50,000–100,000 synchronized animals in L4 to young adult stages were crushed approximately 10 times with liquid N_2_ on a mortar with pestle and resuspended (100 μl) in a buffer that contained 220 mM of manitol, 70 mM of sucrose, 5 mM of MOPS, 2 mM of EGTA and 0.4% BSA, pH 7.4. The homogenized animals were centrifuged at 2,700 rpm for 5 min, supernatant was collected in a separate tube, the pellet was resuspended in previously described buffer and centrifuged again. This procedure was repeated two more times. Collected supernatant was centrifuged at 10,500 rpm for 5 min to obtain the mitochondrial pellet. Mitochondria were resuspended in 250 mM of sucrose and 1 mM of MgCl_2_. We extracted F_1_F_o_-ATP synthase with digitonin 0.66mg/mg of protein, incubate on ice for 10 min and centrifuge for 5 min at 75,000 rpm (Airfuge Ultracentrifuge, Beckman Coulter). The supernatant was used for protein determination and 100 μg of protein was loaded into a first dimension (1D) blue native PAGE [39] followed by a second dimension (2D) SDS-PAGE [40]. For BN-PAGE the digitonin extracted protein was combined with 10 μl buffer 3X (1.5M 6-aminocaproic acid, 150 mM Bis-Tris) plus serva blue G (7 μg/μl stock serva blue G in 0.5M 6-aminocaproic acid) (final concentration, 30 ng of serva Blue G/1 μg of protein), the samples were charged into a BN-PAGE 3.5–11%, and the electrophoresis was performed at 70V for 30 min and 100V for 2h at 4°C. 1D gel was stained with Coomassie solution (0.1% Coomassie Blue R5-250, 45% of methanol and 10% of glacial acetic acid) and stirred at room temperature for 2 h and discolored with a solution containing 30% methanol, 10% glacial acetic acid. Densitometric analysis of dimeric and monomeric bands of each genotype was performed using Fiji (Image J) software. ATPase activity was identified by incubating the first dimension gel in a pre-incubation solution containing 35 mM Tris, 250 mM glycine, pH 8.3. Then, the gel was stirred and incubated for 2 h at 37°C with 5 mM ATP, 5 mM MgCl_2_, 0.15% (w/v) lead acetate, and 150 mM glycine, pH 8.3. After that, the gel was stirred and incubated at room temperature for 24 h. Monomer and dimer bands with dark background were scanned at 2 h.

For 2D SDS-PAGE, the Coomassie gel was denatured with a solution containing 1% SDS, 5 mM DTT for 1 h. The band that corresponds to the dimer and monomer were cut out of the Coomasie gel. The band was rotated 90° on a 15% SDS-PAGE Von Jagow and the electrophoresis was performed at 70V for 30 min and 100V for 2h at 4°C with slight modifications to the described in [41]. The gel was scanned and analyzed.

### Survival assays

Heat shock experiments were performed as previously described [25], with some modifications. Synchronized animals (30–40 animals per plate) were grown at 20°C until animals reached a 4-day-old adult stage, and transferred to several (35 mm) NGM plates seeded with *E*. *coli* OP50 for heat shock. The plates were incubated for up to 12 h at 35°C, and every 2 h the plates were taken from the incubator to observe animals under the stereoscopic microscope. Animals that did not respond to touch were scored as dead.

We tested different concentrations for sodium cyanide (NaCN, 50–100 mM) and carbonyl cyanide m-chlorophenyl hydrazone (CCCP, 50–125 μM). We chose CCCP concentrations that did not affect considerably wild-type nematodes survival but did affect *mai-2* mutant animals. Incubations and recovery were done as previously described [42], with some modifications. One-day-old adult animals (approximately 30) were placed in 200 μl of M9 (control) and M9-containing freshly diluted NaCN or CCCP at indicated concentrations for 1 h at 20°C on a crystal Petri dish. Cyanide when diluted in a buffered solution at pH 7 is known to outgas to hydrogen cyanide (HCN) [43], it has also shown that HCN contributes to the survival of the animal [44]. After the incubation period, 2 ml of M9 were added to dilute the solutions; animals were then recovered by pipetting and placed on *E*. *coli* seeded NGM plates. After the plates were drained from excess liquid for approximately 5 min, the animals were allowed to recover for another 55 min more at 20°C. Finally, the animals were observed under the stereoscopic microscope. Animals that did not respond to touch were scored as dead.

Oxidative stress experiments were performed as previously described [45], with some modifications. Previously, it has been recommended to use a paraquat concentration between 100 mM and 400 mM due to variation in batch potency [46]. We used paraquat at a concentration of 200 mM [45] diluted in M9 with *E*. *coli* OP50. We incubated animals in agitation a 20°C, at indicated time animals were recovered by pipetting and placed on *E*. *coli*-seeded NGM plates. The plates were drained for excess liquid for approximately 5 min and animals were observed under the stereoscopic microscope. Animals that did not respond to touch or showed movement when exposed to a drop of M9 were scored as dead.

### Apoptosis assays

We crossed *mai-2(xm18*) and *mai-2(xm19)* with MD701 *bcIs39*[*Plim-7*::*ced-1*::*gfp*; *lin-15*(*+*)] strain. We selected L4 animals of each strain and approximately 24 h later we mounted on slides using 2% agarose pads; the cell corpses were then visualized by fluorescence microscopy using a Nikon Eclipse E600 microscope equipped with an AxioCam MRc camera (Zeiss). For stress conditions, such as starvation, oxidative stress, heat shock and UV irradiation (DNA damage) we performed experiments as previously described [32,42]. To quantify apoptosis in the soma, synchronized L1 animals of N2, *mai-2(xm18)* and *mai-2(xm19)* strains grown at 20°C were fed *ced-1* dsRNA until they had offspring as previously described [42]. Late stage embryos and L1 larvae were mounted on agarose pads and cell corpses were quantify under Nomarski microscopy.

### Statistical analysis

Brood size, embryonic lethality, TMRM fluorescence (embryos and whole animal), oxygen consumption rates, ATP quantification and germ cell apoptosis (physiological conditions and stress) were tested for equality of group variance. One-way ANOVA followed by Tukey method were performed for those datasets that had the same variance, while for unequal variances, non-parametric comparisons were performed using Dunn's method, with wild-type as a control. *t*-test was used to analyze dimer/monomer F_1_F_o_ATPase ratio. For survival assays two-way ANOVA analysis with Bonferroni test was used to compare each condition to the control. Data was analyzed using GraphPad Prism Statistical Software.

## Results

### MAI-2 is expressed in the mitochondria of *C*. *elegans*

Ichikawa *et al*. previously showed MAI-1 and MAI-2 sub-cellular localization using yeast and demonstrated that MAI-2 is associated with yeast mitochondria, while MAI-1 is not [19]. Because these proteins were not expressed in their natural environment, we determined MAI-1 and MAI-2 expression in *C*. *elegans* and tested whether, in the nematode, MAI-1 could indeed associate with mitochondria. To test this hypothesis, we generated transgenic animals that express Green Fluorescent Protein (GFP) in the C-terminus of the MAI-2 protein (Fig 1C, line 5) (see Materials and methods).

**Fig 2:**
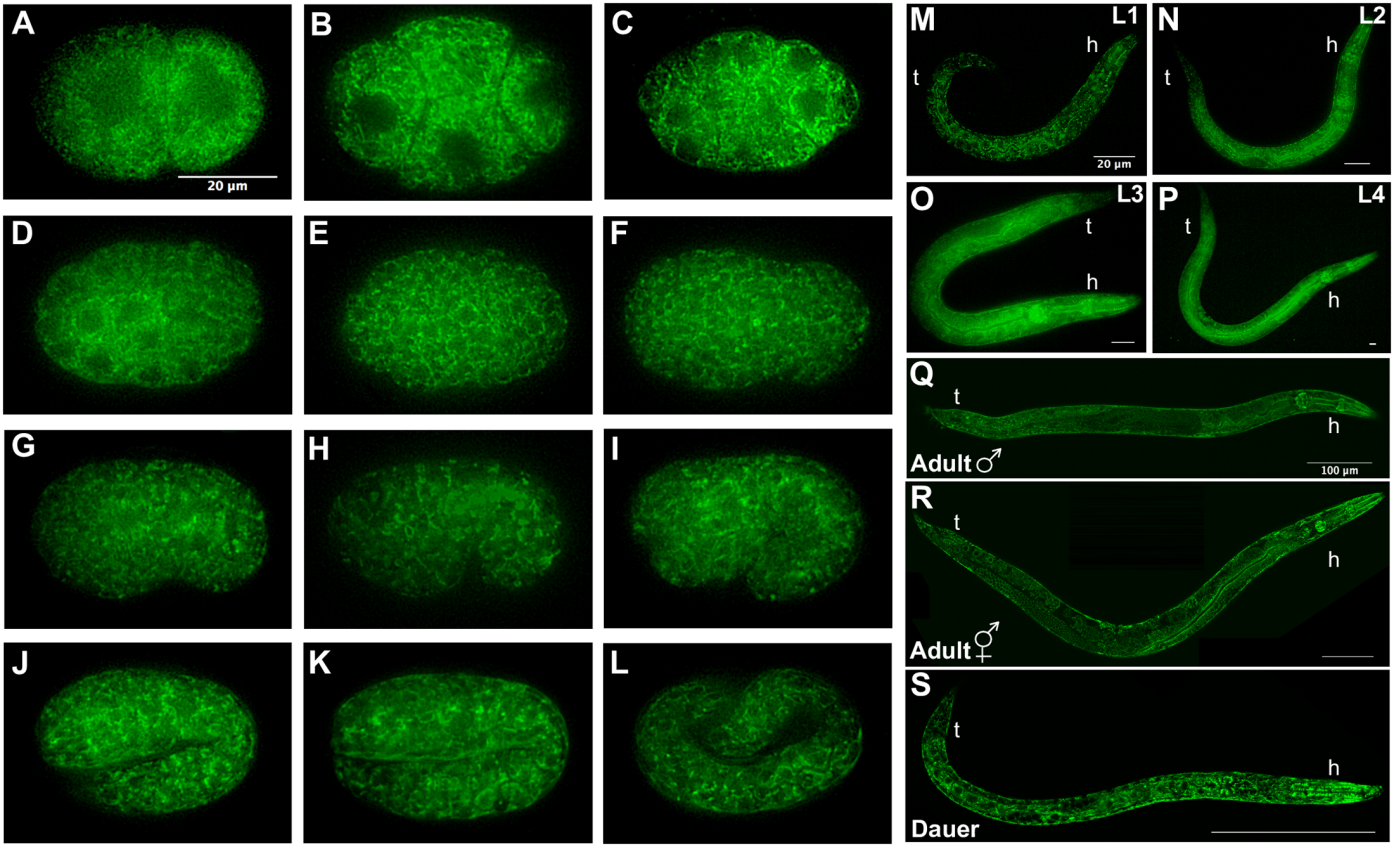
MAI-2::GFP is expressed in the soma and germline. Embryos, larvae and adults expressing the transgene *Pmai-2*::*mai-2*::*gfp*::*mai-2-*3’UTR were observed under a fluorescence microscope. Embryos (A-L), 2-cell (A), 4-cell (B), ~26-cell (C), ~44-cell (D), bean stage (G), comma stage (H), 1.5-fold (I), 2-fold (K), 3-fold (L). Indicate larval stages (M-P). Male adult (Q). Hermaphrodite adult (R). Dauer larva (S). h: head, t: tail.

A mix of MAI-2::GFP embryos, synchronized hermaphrodite larvae (L1-L4), one-day-old hermaphrodite and male adults, and dauer larvae were mounted and observed under the fluorescence microscope. We observed the expression of the transgene in the cytoplasm at all the embryonic (Fig 2A–2L) and post-embryonic stages (Fig 2M–2S) in what seems to be a mitochondrial pattern. The expression of MAI-2::GFP was observed in the germline and all somatic tissues, including neurons, pharynx, the intestine, body wall muscle and the hypodermis, during *C*. *elegans* development.

**Fig 3:**
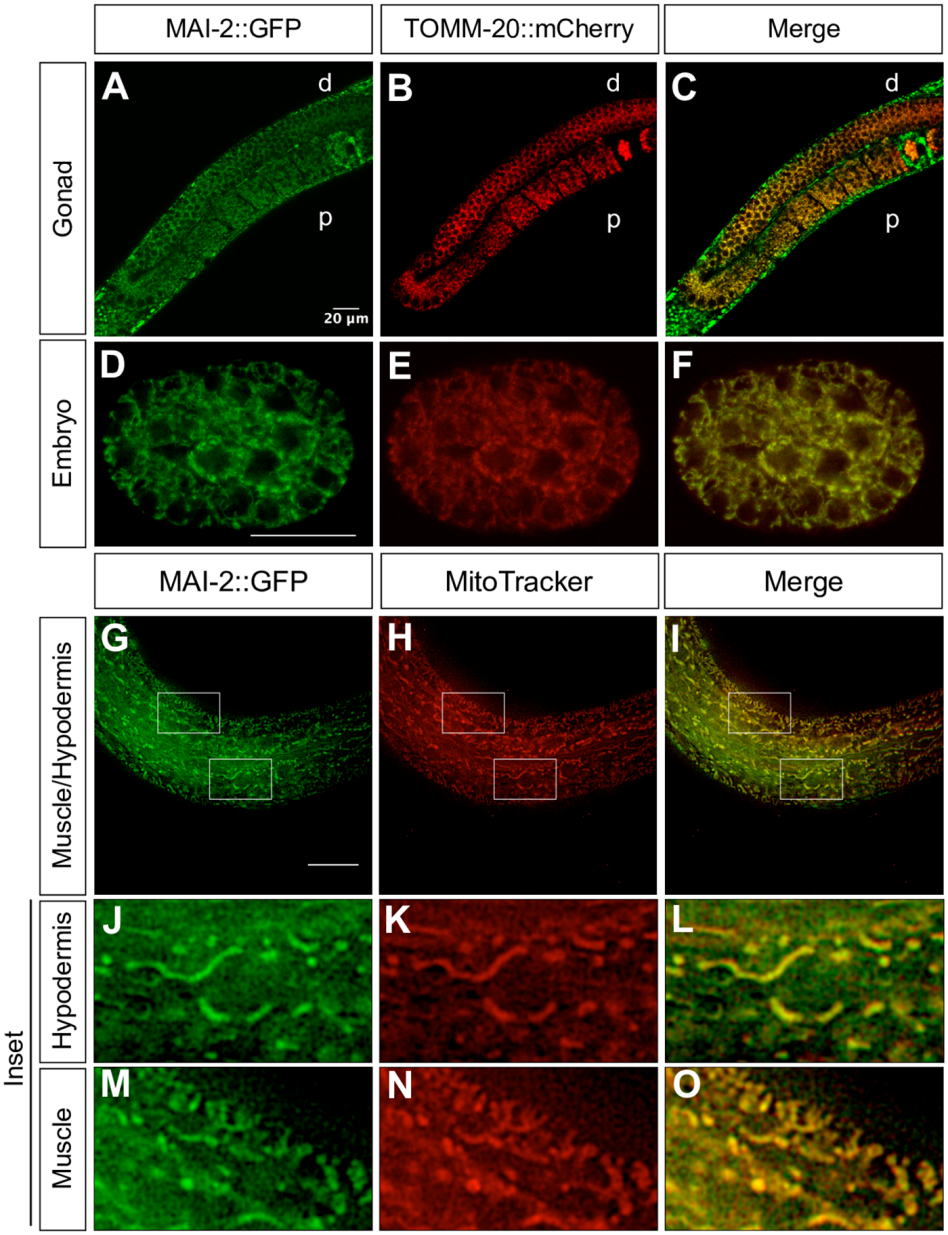
MAI-2::GFP is localized in the mitochondria. We crossed MAI-2::GFP animals with mitochondria expressing transgenic TOMM-20::mCherry animals (A-F). We mounted and observed one-day-old adult animals under a confocal microscopy and observed co-localization in germ cells (A-C) and embryos (D-F). Stained MAI-2::GFP animals with MitoTracker Red CMXRos were mounted and observed under a fluorescence microscope (G-O). We observed the co-localization of signals in tissues such as the hypodermis (J-L) and muscle (M-O), shown in the inset. d: distal, p: proximal.

To corroborate MAI-2::GFP mitochondrial localization in *C*. *elegans*, we crossed the MAI-2::GFP transgenic with mitochondrial protein reporter transgenic animals *xmSi01*[*Pmex-5*::*tomm-20*::*mCherry*::*tbb-2* 3’-UTR; *Cbr-unc-119* (+)]II and looked for co-localization under normal conditions (Fig 3). The *mex-5* promoter region drives TOMM-20::mCherry expression in the germ cells and embryo [47]. We found that MAI-2::GFP co-localized with mitochondria in the gonad (Fig 3A–3C) and embryos (Fig 3D–3F). To further corroborate MAI-2::GFP expression in mitochondria, we also stained the transgenic MAI-2::GFP animals with the mitochondrial probe MitoTracker Red CMXRos (Fig 3G–3O). For this purpose, one-day-old adult worms were incubated in M9 with MitoTracker Red CMXRos and were observed under a fluorescence microscope. Mitotracker Red CMXRos has been used to stain mitochondria however works better in mitochondria of the body muscle cells [34] and the hypodermis [33]. We found a clear co-localization of MAI-2::GFP with the mitochondrial probe in the hypodermis (Fig 3J–3L) and muscle (Fig 3M–3O). These results demonstrate that MAI-2::GFP is a mitochondrial protein.

**Fig 4:**
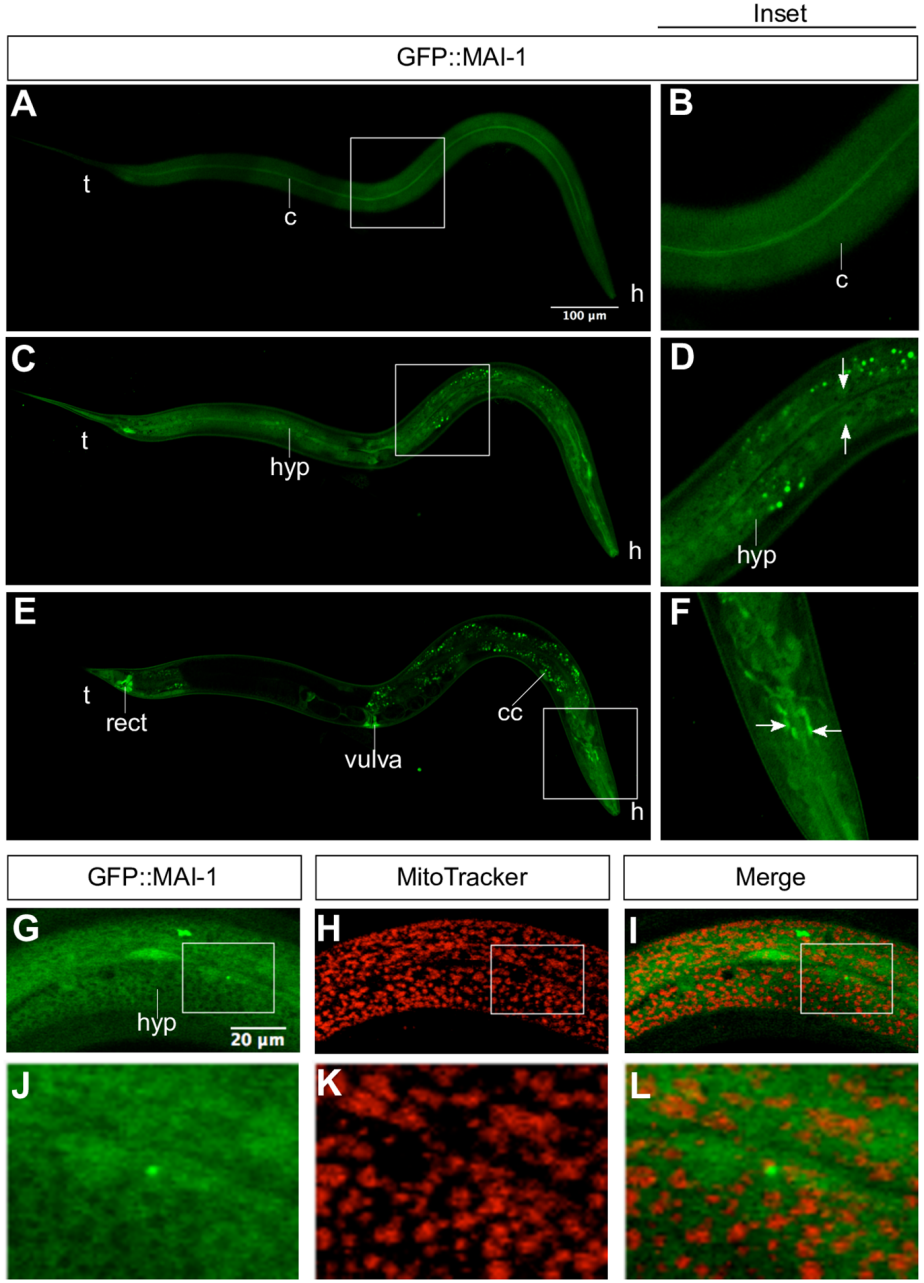
GFP::MAI-1 is diffusely expressed in the cytoplasm and some nuclei. We mounted one-day-old adult animals expressing the transgene *Pmai-1*::*gfp*::*mai-1-*3´UTR and observed them under a confocal microscope. We observed the expression of GFP::MAI-1 in the cuticle (A-B), cytoplasm and nuclei (arrow) of the hypodermis (C-D), in the rectum, vulva, and coelomocytes (E) and in some neurons of the animal´s head (E,F). Details of each picture are shown in the right (inset). We stained GFP::MAI-1 animals with MitoTracker CMXRos and observed no co-localization between them (G-I). Details of each picture are shown on the bottom (J-L). h: head, t: tail, c: cuticle, hyp: hypodermis, rect: rectum, coelomocytes: cc.

To study MAI-1 expression in *C*. *elegans*, we generated by MosSCI, N-terminal GFP and C-terminal mCherry protein reporter transgenes (Fig 1C, line 6 and 7) (see Materials and methods). GFP::MAI-1 (*xmSi32*) and MAI-1::mCherry (*xmSi31*) showed the same tissue localization (Fig 4; S1 Fig). GFP::MAI-1 is expressed in specific somatic tissues in the nuclei and in the cytoplasm through specific tissues, including the cuticle (Fig 4A and 4B), hypodermis (Fig 4C and 4D), rectum, vulva and neurons (Fig 4E and 4F). Although the expression pattern did not suggest a mitochondria network and MAI-1 does not carry a mitochondrial transport sequence, we tested whether MAI-1 was expressed in the mitochondria by incubating GFP::MAI-1 with Mitotracker Red CMXRos and found no co-localization between the mitochondrial probe and GFP::MAI-1 (Fig 4G–4L). These results demonstrate that MAI-1 is indeed not expressed in mitochondria but in the cytoplasm, as previously suggested Ichikawa *et al*. Additionally, we observed that GFP::MAI-1 is expressed in the nuclei. Because MAI-1 does not seem to be a canonical IF_1_, we studied only MAI-2.

### *mai-2* mutant animals show no defects under physiological conditions

**Fig 5:**
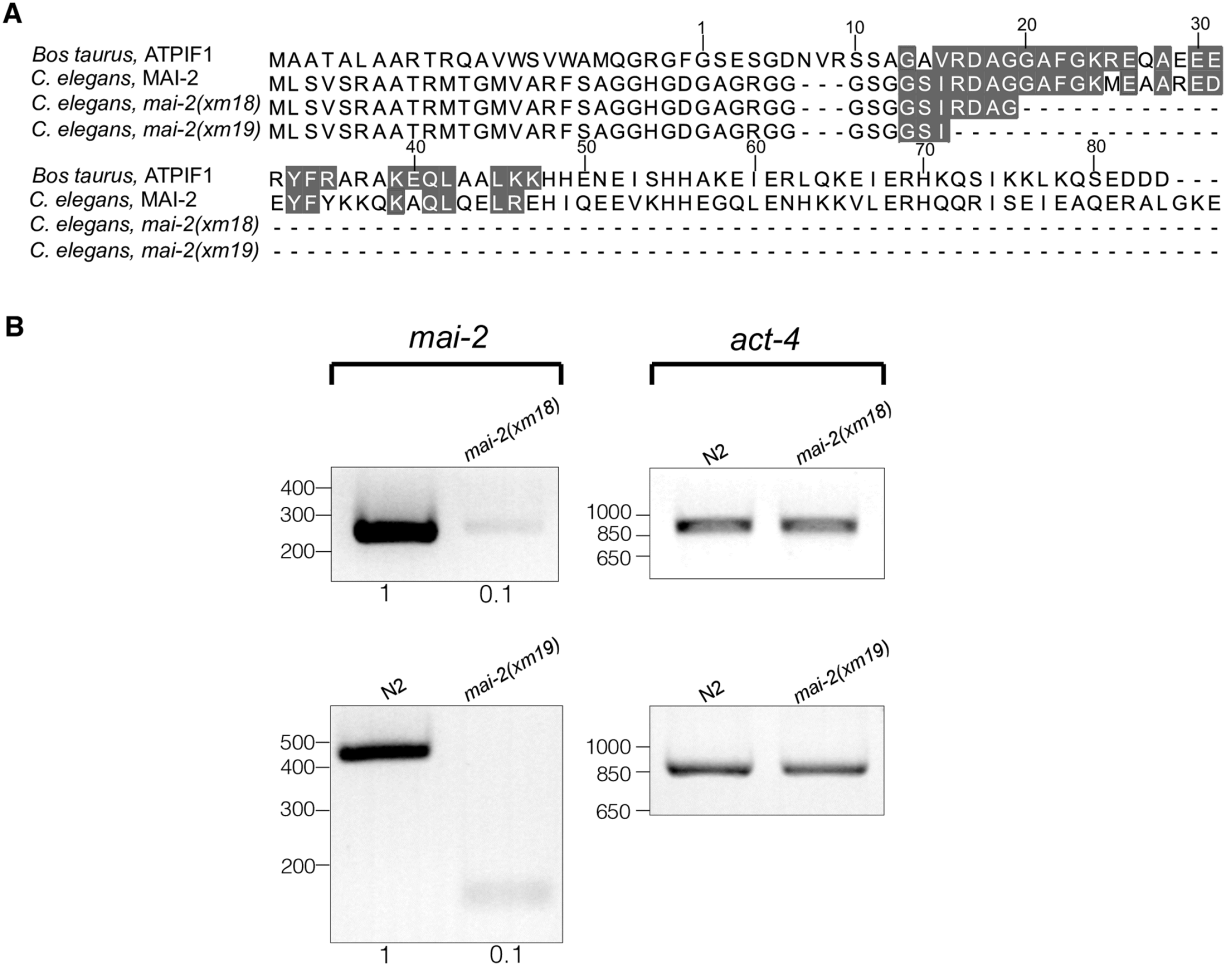
*mai-2* mRNA is barely detectable in *mai-2* mutants. (A) The amino acid sequences of wild-type and putative MAI-2 mutant proteins (alleles *xm18* and *xm19*) of *C*. *elegans* are compared with the amino acid sequence of *Bos taurus*. The residues that form the minimal inhibitory sequence are shaded in gray [19]. (B) Semi-quantitative RT-PCR analysis using cDNA synthesized from RNA extracts obtained from one-day-old control animals (N2) and *mai-2* mutant animals (alleles *xm18* and *xm19*). Specific primers for *mai-2* and *act-4* were used; *act-4* was used as a loading control. Densitometry analysis was performed, normalizing each band with its corresponding *act-4* band using ImageJ software.

**Fig 6:**
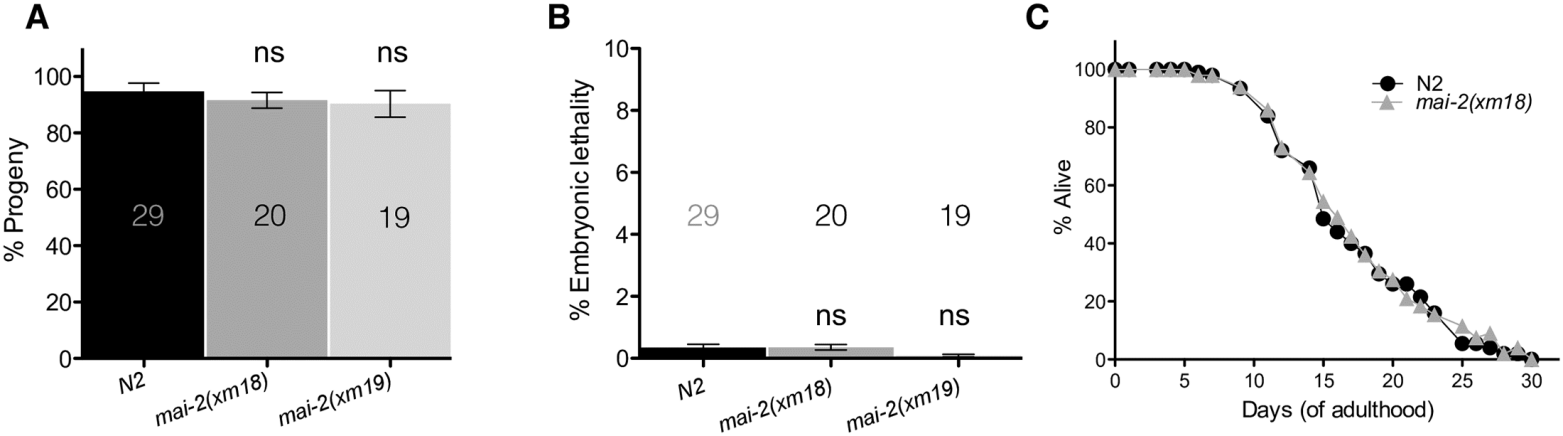
*mai-2* mutants show no defects in fertility or lifespan. To quantify the offspring and embryonic development, L4 wild-type (control), *mai-2(xm18)* and *mai-2(xm19)* animals were transferred to new plates every 24 h over the course of 4 days at 20°C. The plates were scored for offspring (A) and dead embryos (B). Total progeny included all the offspring that developed (as larvae) or died as embryos. Embryos not hatching after 24 h after being laid were considered dead. For N2, the average of three independent experiments is shown, for *mai-2(xm18)* and *(xm19)* the average of two independent experiments is shown (means±SEM). Statistical significance was determined by one-way ANOVA followed by Tukey post-test and was not significant (ns). The number of analyzed animals (N) is indicated in each graph. (C) For the lifespan assay, wild-type and *mai-2(xm18)* animals were scored every other day until all of them were found death (aprox. 30 days) at 20°C. The data represent the mean of two independent replicates in which 50 worms per group were assayed. The interaction effect of wild-type and *mai-2(xm18)* was calculated by two-way ANOVA and were not significant (not shown).

To study the function of *mai-2*, we generated mutants by CRISPR/Cas9 genome editing (Fig 1A, line 1 and 2) [29,30], see Materials and methods). Allele *mai-2(xm18)* contains an insertion of a PvuII restriction enzyme site, resulting in a premature stop codon that produces a truncated MAI-2 protein of 41 amino acids (Fig 1A and 1B, line 1 and 3; Fig 5A). The allele *mai-2(xm19)* carries a 794-bp deletion consisting of the following: 151 bp (from 261 bp) of the first exon, the entire intron (525 bp), exon 2 (69 bp) and 49 bp from the *mai-2* 3’-UTR, resulting in a putative truncated MAI-2 protein of 37 amino acids (Fig 1A and 1B, line 2 and 4; Fig 5A). We tested the abundance of the *mai-2* mRNA by semi-quantitative RT-PCR in *mai-2(xm18)* and *mai-2(xm19)* mutant backgrounds. We found that the mRNA in both mutants was down-regulated by 90% (Fig 5B). These results confirmed that the mRNA encoding the predicted truncated proteins in both *mai-2* mutants are present in low abundance because it might be degraded by the nonsense-mediated decay machinery [48]. *mai-2* mutant animals showed no evident phenotype under normal conditions, had normal fertility (tested in *xm18* and *xm19* strains) (Fig 6A), no embryonic lethality (tested in *xm18* and *xm19* strains) (Fig 6B), and normal lifespan (tested only in *xm18*) (Fig 6C).

### *mai-2* mutants animals show no defect in the mitochondria network and ATP synthase dimerization but show higher Δ*ψ*_m_

**Fig 7:**
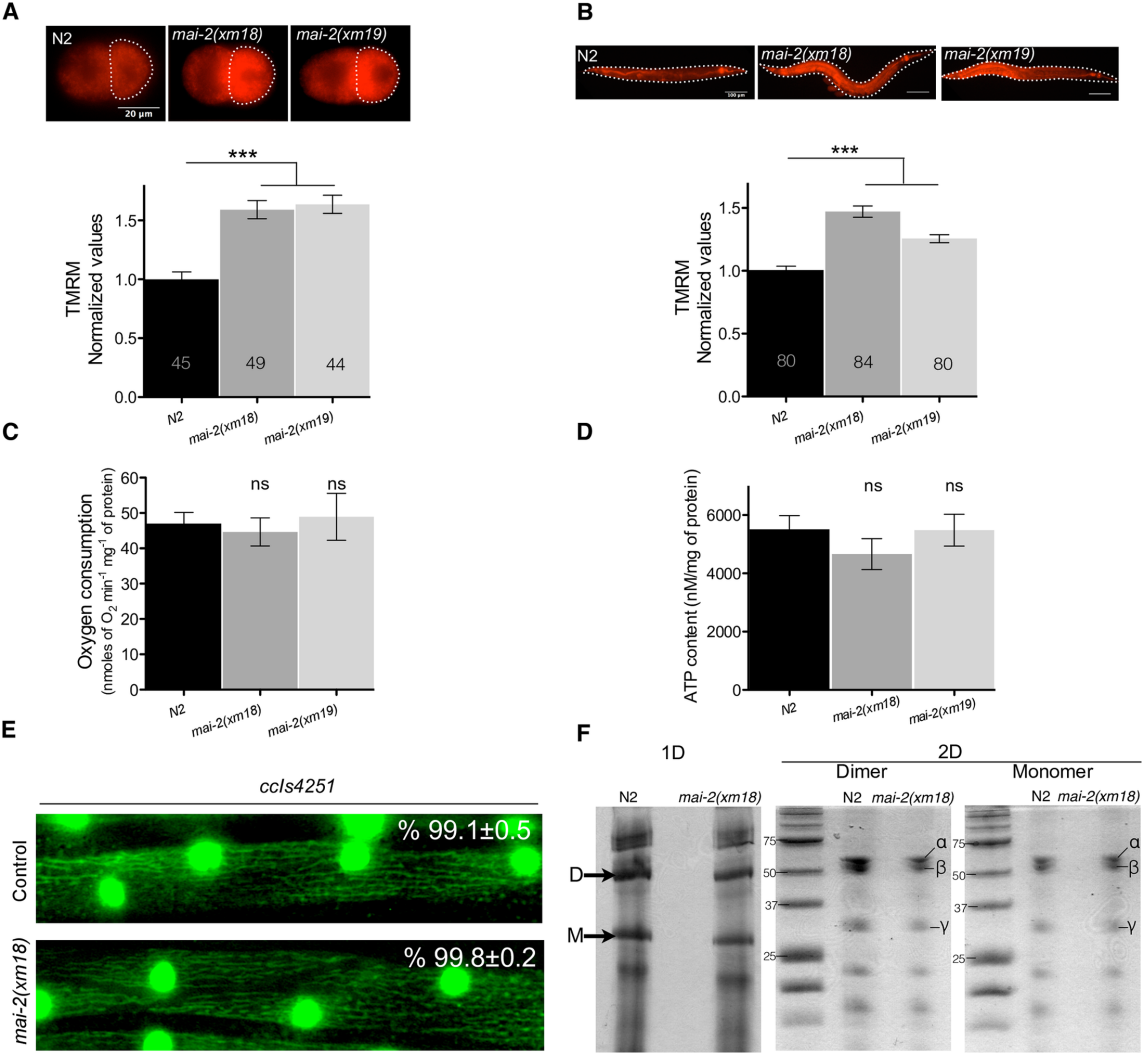
Δ*ψ*_m_ is elevated in *mai-2* mutants. (A-B) To test the Δ*ψ*_m_ of one-day-old N2, *mai-2(xm18)* and *mai-2(xm19)* synchronized adults were grown in NGM with TMRM for approximately 15 h, after incubation animals were dissected for embryo extraction. 2-cell embryos and the whole animal were observed under a fluorescence microscope. The fluorescence of TMRM (arbitrary units) was quantified for each of the genotypes and conditions. The values were normalized relative to the fluorescence of wild-type animals, grown at 20°C in normal conditions. For N2 embryos, the average of four independent experiments is shown, four for *mai-2(xm18)* and two for *mai-2(xm19)*. For whole animals, the average of six independent experiments for each indicated background is shown. The bars show mean±SEM and inside each bar the number of animals tested is indicated. The *** (*P*<0.0001) symbols placed on top of the bars are comparisons between wild-type and *mai-2* mutants, statistical significance was determined by Dunn´s method. (C) To quantify oxygen consumption, a pool of L4 to early adults was introduced in a water-jacketed chamber and oxygen consumption rate was recorded until linear. Oxygen consumption was normalized with protein concentration. Average of four independent experiments for N2, four for *mai-2(xm18)* and two for *mai-2(xm19)* is shown. (D) For ATP quantification, ATP was extracted from 50 one-day-old adult animals and was quantified with a bioluminescent assay. ATP content was normalized by protein concentration. The average of four independent experiments of each indicated background is shown. (C-D) The bars indicate mean±SEM. The ns (non-significant) symbols placed on top of the bars are comparisons between wild-type and *mai-2* mutants, statistical significance was determined by one-way ANOVA followed by Tukey post-test. (E) L4 animals of the strain SD1347 *ccIs4251*[*myo-3p*::GFP(NLS)::LacZ (pSAK2)+*myo-3p*::GFP (mitochondrially targeted) (pSAK4)+dpy-20(+)] and SD1347 crossed with *mai-2(xm18)* were mounted on agarose pads and observed under a fluorescence microscope. The mitochondria network morphology was assessed in the body wall muscle and the percentage of cells with tubular mitochondria was determined from two independent experiments. A representative picture of each condition is shown. The percentage of tubular mitochondria and SEM are shown. (F) Purified F_1_F_o_-ATP synthase from *C*. *elegans* control and *mai-2(xm18)* animals were subjected to 1D BN-PAGE and 2D SDS-PAGE. The arrows in 1D gel indicate the dimeric (D) and monomeric (M) bands. The 2D gel shows the F_1_F_o_-ATP synthase subunits (α, β, γ) which constitutes, among other subunits, the dimer and the monomer of N2 and *mai-2(xm18)* animals. The data of one of three independent experiments are shown.

We studied the effect of *mai-2* mutation in the Δ*ψ*_m_ of the mitochondria of *C*. *elegans* by incubating animals in NGM-lite plates with the TMRM fluorescent probe. These compounds accumulate inside the mitochondria of the embryo proportionally to the mitochondrial Δ*ψ*_m_ [49]. We measured the intensity of TMRM fluorescence on the P_1_ cell of 2-cell embryos and in the complete animal under normal conditions (Fig 7A and 7B). We found that *mai-2(xm18)* and *mai-2(xm19)* animals generated higher Δ*ψ*_m_ than wild-type animals.

Due to the high Δ*ψ*_m_ observed in our experiments, we evaluated the animal respiration of the wild-type and *mai-2* mutants. Synchronized L4 to early adult animals grown in NGM-lite and *E*. *coli* OP50 bacteria were collected, washed and introduced into a water-jacketed chamber, and the oxygen consumption rate was recorded until the trend was linear (resting state) [50]. Despite the high Δ*ψ*_m_ previously observed, we found that *mai-2(xm18)* and *mai-2(xm19)* showed no differences in oxygen consumption compared with wild-type animals (Fig 7C). To evaluate whether the increase in Δ*ψ*_m_ was a consequence of an increase in hydrolysis, we analyzed the total [ATP] of whole animals and observed a decrease of ~15% in *mai-2(xm18*) (Fig 7D); however, this difference was non-significant. These results indicate that, in the absence of MAI-2 protein, the electron transport chain may not be disturbed and Δ*ψ*_m_ could be affected by another mechanism.

To determine whether, in *C*. *elegans*, the absence of *mai-2* may promote changes in mitochondrial morphology, we crossed *mai-2(xm18)* animals with SD1347 *ccIs4251* I. This transgene expresses GFP in the mitochondria networks of the body wall muscle, which carry a nuclear- and mitochondria-targeted GFP under the control of the *myo-3* promoter [51]. We observed that L4 control animals displayed mostly tubular mitochondria (99.1%). *ccIs4251* I*;mai-2(xm18)* revealed no evident defects in the mitochondria network and showed mostly tubular mitochondria (99.8%) (Fig 7E). For N2 animals, 555 cells of 22 animals were visualized while 612 cells of 27 animals were visualized for *mai-2(xm18)*. Furthermore, we studied in *mai-2(xm18)* ATP synthase dimerization using blue native gels. We observed that, in *mai-2(xm18)*, ATP synthase dimers, with respect to the monomer content of ATP synthase dimers, were unmodified when compared with control animals [N2 = 0.95±0.12, *mai-2(xm18)* = 0.99±0.07. Data represents means±SEM and comparisons were non-significant after *t*-test analysis] (Fig 7F). To demonstrate that the dimer and monomer bands corresponds to F_1_F_o_-ATP synthase, we analyzed by a 2D SDS-PAGE F_1_F_o_-ATP synthase subunits content [40] and we clearly distinguished the (α, β and γ) subunits. We further corroborated that we were observing the dimer and monomer forms of the F_1_F_o_-ATP synthase by a blue native gel to detect ATPase activity (S2 Fig).

### MAI-2 is required for stress survival

**Fig 8:**
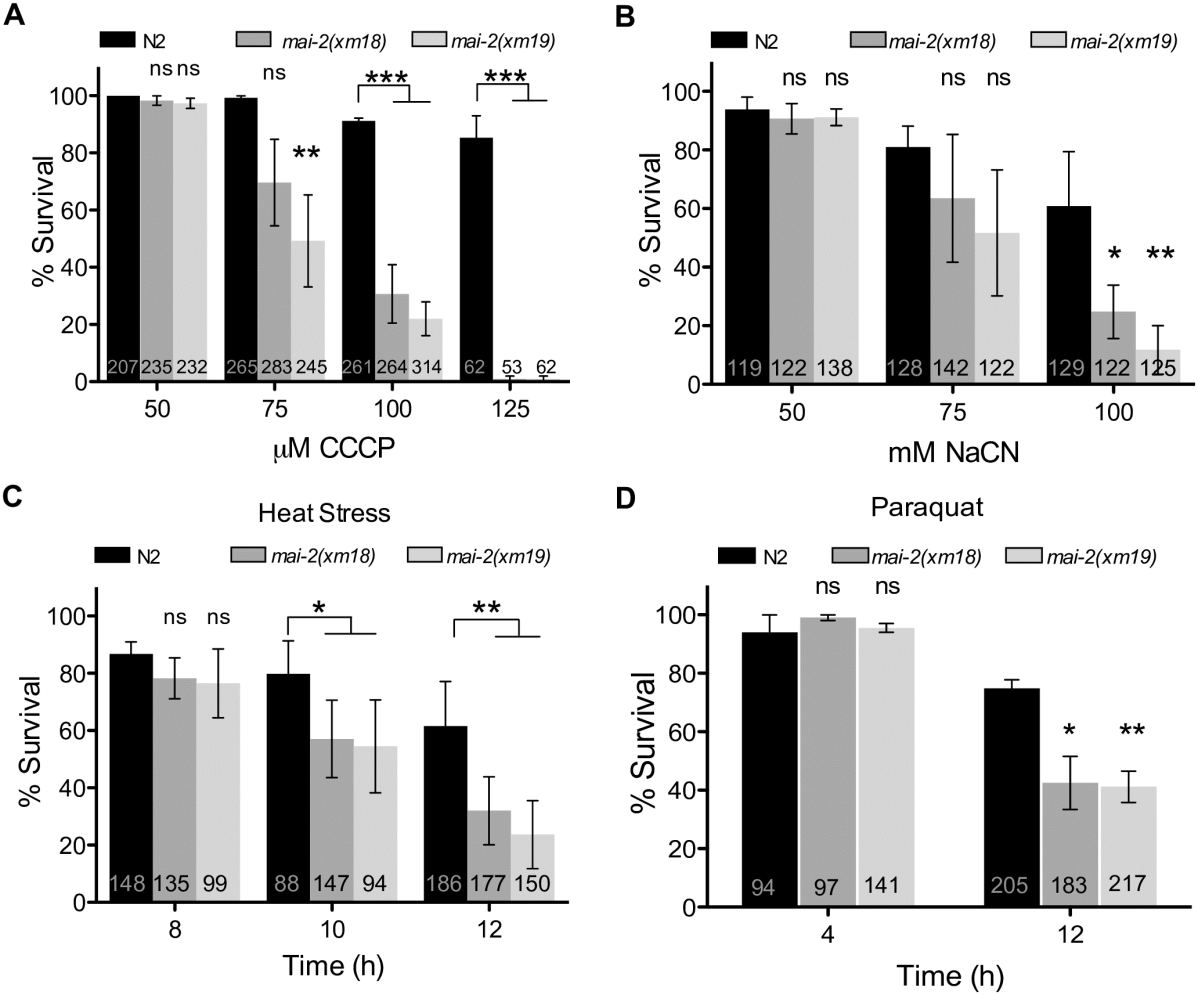
MAI-2 is required for stress survival. We exposed synchronized one-day-old adults N2, *mai-2(xm18)* and *mai-2(xm19)* to different concentrations of CCCP (A) and NaCN (B) for 1 h and recover them on NGM plates seeded with bacteria for 1 h. For CCCP experiments the average of four independent experiments is shown, while for NaCN experiments we show the average of three independent experiments. (C) We heat shocked synchronized 4-day-old adults of the indicated background at 35°C for 12 h; every 2 h animals were observed under the dissecting scope for animal survival. For N2 and *mai-2(xm18)* we show the average of four independent experiments, while for *mai-2(xm19)* we show the average of two independent experiments. (D) We incubated one-day-old adults of the indicated background in 200 mM paraquat and scored for survival every 4 h through an interval of 12 h. We show the average of three independent experiments. (A-D) Animal survival was quantified by the lack of movement when touched. The data represent means±SEM and inside each bar the number of animals tested is indicated. Two-way ANOVA followed by Bonferroni post-test was used to compare each condition with the control, ns represents non-significant. ***P<0.001, **P<0.01, *P<0.05.

To examine the role of MAI-2 in the whole animal survival under stress conditions that affect the mitochondria, we exposed control and *mai-2* mutant one-day-old hermaphrodite adults to different concentrations of the respiratory chain inhibitor sodium cyanide (NaCN) and the uncoupler carbonyl cyanide *m*-chlorophenyl hydrazone (CCCP) for 1 h and then quantified the animals' survival. We found that *mai-2(xm18)* and *mai-2(xm19*) were not sensitive to NaCN or CCCP compared with control animals at concentrations less than 75 mM or 75 μM, respectively; however, at higher concentrations, *mai-2* mutants were more sensitive and died at a higher percentage than wild-type animals (Fig 8A and 8B).

High temperatures increased ATP depletion [31]; therefore, we heat shocked 4-day-old control and *mai-2* hermaphrodite animals for 12 h at 35°C. We analyzed the mortality every 2 h. During the first 8 h of incubation, we observed no significant difference between control and *mai-2* mutant animals (Fig 8C). However, at 12 h of heat shock, we observed that 60% of *mai-2* mutants were dead compared with control animals (Fig 8C).

To determine whether MAI-2 participates in oxidative stress resistance, we incubated *mai-2* mutants and control adult animals in the presence of 200 mM paraquat, a poison that promotes ROS production for 12 h. We observed that *mai-2* mutants died 30% more than control animals at 12 h of incubation with paraquat (Fig 8D), demonstrating that they are more vulnerable to oxidative stress. These data support that *mai-2* mutants are less resistant to stress.

### MAI-2 is required for stress-induced germ cell apoptosis

**Fig 9:**
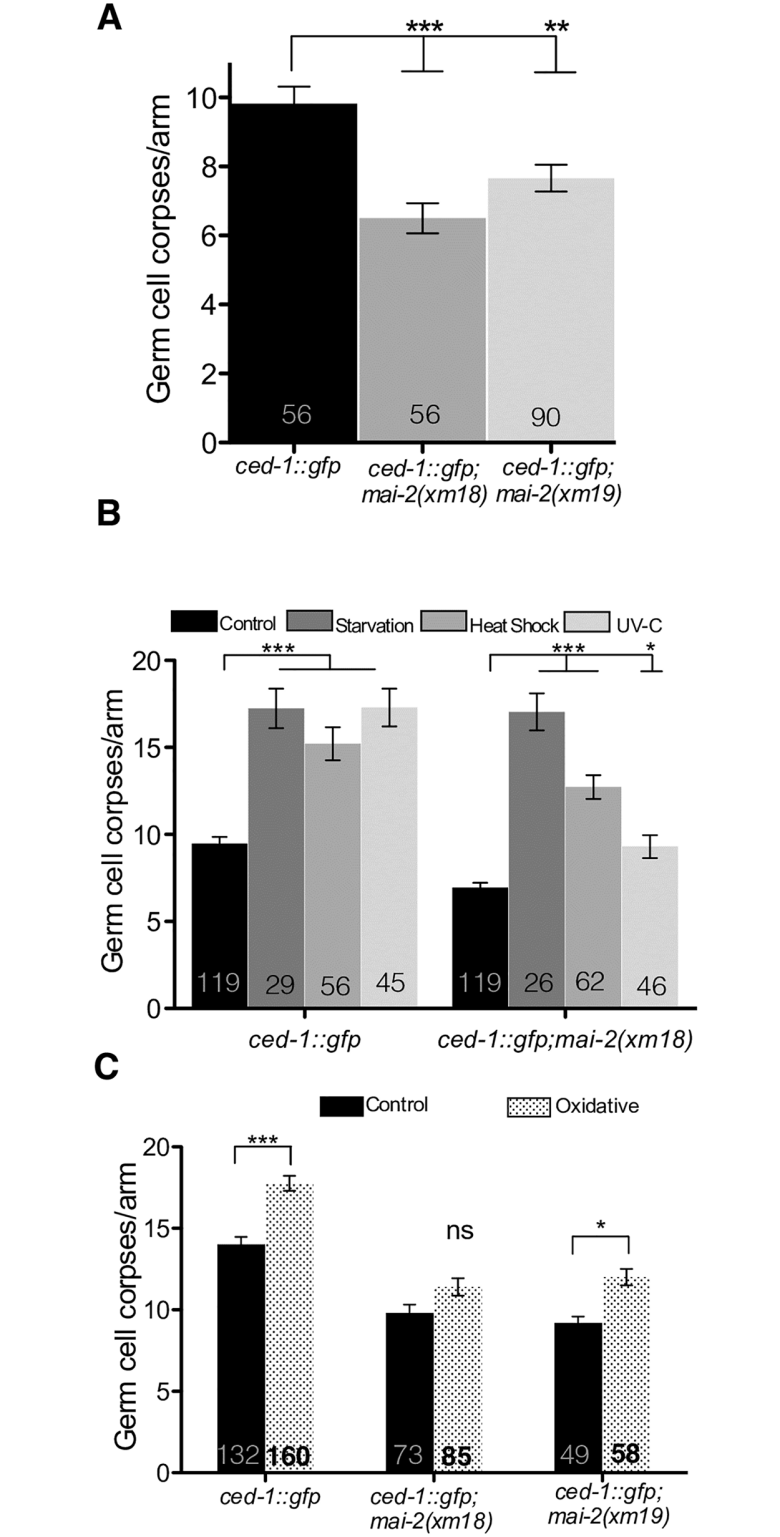
MAI-2 is important to induce apoptosis under physiological and stress conditions. Germ cell apoptosis was assessed in *mai-2* mutants using the strain MD701 *bcIs39*[*Plim-7*::*ced-1*::*gfp; lin-15*(+)] in which apoptotic cell corpses can be easily visualized under a fluorescence microscope. Germ cell corpses per gonad arm were counted in one-day-old adults of the indicated backgrounds under physiological (A) and stress conditions (B-C). Animals were exposed to starvation, heat shock, UV-C irradiation (B) and paraquat (C), as indicated in the materials and methods section. We show the average of three independent experiments for physiological apoptosis (A). For apoptosis induced by starvation, heat shock and UV-C irradiation we show the average of two independent experiments (B), while for apoptosis induced by oxidative stress we show the average of three independent experiments for *ced-1*::*gfp* and *ced-1*::*gfp;mai-2(xm18)* mutant, while the average of two independent experiments are shown for *ced-1*::*gfp;mai-2(xm19)* (C). (A-C) The data represent means±SEM and the number of animals analyzed in each condition is shown in each bar. Dunn´s test was used to compare each condition with the control and ns represents non-significant. ***P<0.0001, **P<0.001, *P<0.01.

To explore the effect of *mai-2* mutation over apoptosis on *C*. *elegans*, we examined somatic (embryogenesis), physiological and stress-induced germ cell apoptosis (gonads) in wild-type and *mai-2* mutants. For apoptosis quantification, we crossed the strain MD701, which is commonly used to quantify apoptotic corpses using fluorescence microscopy [52], with *mai-2(xm18)* and *mai-2(xm19)* animals. *ced-1*::*gfp* control animals, and *ced-1*::*gfp;mai-2(xm18)* and *ced-1*::*gfp*;*mai-2(xm19)* mutant animals were grown at 24°C and were observed under a fluorescence microscope to quantify cell corpses at 24 h post L4. We found that *ced-1*::*gfp*;*mai-2(xm18)* and *ced-1*::*gfp*;*mai-2(xm19)* showed a significant decrease in physiological germ cell apoptosis (Fig 9A). By contrast, we found no differences in somatic apoptosis between wild-type and *mai-2* mutant animals when we quantified cell corpses during the late stages of embryogenesis and early larval stages [Bean embryo stage: *ced-1(RNAi)* = 1.8±0.1 cell corpses; *mai-2(xm18); ced-1(RNAi)* = 1.6±0.1 cell corpses; *mai-2(xm19); ced-1(RNAi)* = 1.6±0.1 cell corpses. Pretzel embryo stage: *ced-1(RNAi)* = 2.7±0.1 cell corpses; *mai-2(xm18); ced-1(RNAi)* = 2.5±0.1 cell corpses; *mai-2(xm19); ced-1(RNAi)* = 2.6±0.1 cell corpses. L1 larval stage: *ced-1(RNAi)* = 1.4±0.1 cell corpses; *mai-2(xm18); ced-1(RNAi)* = 1.4±0.1 cell corpses; *mai-2(xm19); ced-1(RNAi)* = 1.4±0.1 cell corpses].

We tested the role of MAI-2 in germ cell apoptosis induced by stress, including starvation, heat shock, UV-C stress and oxidative stress [42]. *ced-1*::*gfp* control animals showed an increase in germ cell apoptosis after starvation (1.8-fold), heat shock (1.6-fold) and UV-C irradiation (1.8-fold) (Fig 9B) of the animals. *mai-2(xm18);ced-1*::*gfp* also showed a significant increase in apoptotic corpses under starvation (2.4-fold), heat shock (1.8-fold) and UV-C stress (1.3-fold) (Fig 9B). Although we observed a significant induction of apoptosis under these stressors, we did not observe the same induction levels, only a partial response, for heat shock and UV-C stress.

To induce oxidative stress, we incubated *ced-1*::*gfp* and *ced-1*::*gfp;mai-2(xm18)* for 1 h in paraquat and allowed them to recover in NGM plates seeded with bacteria for 1 h. After the recovery period, we mounted animals and counted apoptotic corpses under a fluorescence microscope. We found that control *ced-1*::*gfp* animals showed a slight but significant increase in apoptotic corpses, but *mai-2(xm18)* and *mai-2(xm19)* did not show a significant increase or show a partial response in germ cell apoptosis (Fig 9C). Our data showed that MAI-2 is necessary to induce apoptosis in the gonad under physiological conditions and under oxidative stress, heat shock, and UV-C. However, MAI-2 does not play a role during the somatic apoptosis, which occurs during late embryogenesis and the early larval stages.

## Discussion

### MAI-1 and MAI-2 is expressed differentially

In this study, we showed the expression of MAI-1 and MAI-2 in their natural environment and during development. We found that MAI-2::GFP is expressed in the mitochondria of all *C*. *elegans* tissues (Fig 2) through all embryonic and larval stages of development. Although MAI-1 does not have a predictable mitochondrial import signal sequence and Ichikawa *et al*. previously observed that, in yeast, this protein does not associate with mitochondria [19], we could not rule out that certain protein-protein interactions could direct MAI-1 to the mitochondria in the nematode. We found that GFP::MAI-1 fusion protein is indeed not associated with mitochondria and, instead, is expressed in the cytoplasm of specific tissues such as the cuticle, hypodermis, neurons of the head, rectum, coelomocytes and vulva (Fig 4A–4F). Unexpectedly, MAI-1 was also found in the nuclei of certain cells, although it lacks an obvious NES sequence (Fig 4D and S1 Fig), which was not observed when MAI-1 was expressed in yeast [19]. However, we cannot rule out that the endogenous MAI-1 protein might have a different localization because MAI-1 specific antibodies are yet not available. Because of its specific tissue localization and nuclear and cytoplasmic expression, MAI-1 remains an interesting target for future research.

### Under normal conditions, *mai-2* mutants display an increased Δ*ψ*_m_ and less germ cell apoptosis

We found no evident alterations in the phenotype of *C*. *elegans* mutants under normal conditions when we tested the lifespan, fertility and embryonic lethality (Fig 6A–6C). Our work supports observations made by Nakamura *et al*. in which no evident phenotype was observed in IF_1_ mutant mice grown under normal conditions [17]. Furthermore, we observed no defects in the mitochondrial network when we used muscle expressing transgenes in a *mai-2* mutant background (Fig 7E) or any changes in the ATP synthase dimer/monomer ratio (Fig 7F). Although we observed that, in *C*. *elegans*, MAI-2 is not necessary for F_1_F_o_ dimer assembly, it was observed recently that IF_I_-KO mice indeed have abnormal cristae formation and scarcer mitochondria [16].

Δ*ψ*_m_ is important for mitochondria function, and when lost, it affects mitochondria in a lethal way due to the halt of ATP production by F_1_F_o_-ATP synthase, mitochondria dynamics [53] and Δ*ψ*_m_-dependent transport activities [54]. Here, we show that *mai-2* mutants had increase Δ*ψ*_m_. Similarly, Fujikawa *et al*. [12] and Shah *et al*. [13] found higher Δ*ψ*_m_ in IF_1_-KD cells than in control cells. In these studies, they also observed lower ATP levels; therefore, they explained that the increase in Δ*ψ*_m_ was probably due to the high hydrolyzing activity of F_1_F_o_-ATPase. We did not observe any difference in the ATP concentration in MAI-2 deficient nematodes. Our results are related to previous observations in which IF_1_-silenced osteosarcoma cells show higher Δ*ψ*_m_ but no significant decrease in the ATP concentration [15].

Δ*ψ*_m_ loss is associated with apoptosis, although their relationship is not entirely yet understood [55,56]. It is believed that the mitochondrial permeability transition pore (PTP) releases certain mitochondrial apoptogenic factors, which, in turn, trigger apoptosis. The probability of PTP opening increases with a decrease in Δ*ψ*_m_ [55,57]. In our work, we found that *mai-2* mutants had a significantly lowered physiological germ cell apoptosis (Fig 9A). In addition, we observed that *mai-2* mutants had a partial apoptotic response to stresses like heat shock, exposure to UV-C and oxidative stress (Fig 9B and 9C). By contrast *mai-2* mutant animals show no changes in somatic apoptosis. Apoptosis in *C*. *elegans* soma and germline is regulated differentially. Apoptosis in the soma (embryo and larval stages) is regulated by the pro-apoptotic protein EGL-1 (a BH3-only domain protein) [42], while physiological germ cell apoptosis (in the gonad) is induced partially by the nematode Retinoblastome homologue protein LIN-35 [58] independently of EGL-1 [59]. Different pathways induce germ cell apoptosis under stress in *C*. *elegans* [42]. Germ cell apoptosis induced by DNA damage is regulated by the p53 worm homologue CEP-1 [60], which induces EGL-1 expression. Heat shock and oxidative stress induce germ cell apoptosis through the MAPK pathway and independently of EGL-1 [42]. Germ cell apoptosis induced by starvation is regulated by CED-9/BCL-2 down-regulation via LIN-35/RB [61].

We hypothesized that there might be a correlation between the lower germ cell apoptosis and high Δ*ψ*_m_ observed in MAI-2 deficient nematodes. Chen *et al*. proposed that IF_1_ deficiency maintains Δ*ψ*_m_ when complex III is inhibited, promoting cell survival and mitochondrial health [14]. Interestingly, IF_1_ has also been proposed to play an anti-apoptotic role, directly linked to IF_1_'s role in regulating mitochondria cristae structure and the mitochondrial network integrity. Faccenda *et al*. showed that IF_1_-KD have cristae defects that enhanced cytochrome *c* liberation and the apoptotic cascade when treated with staurosporine [11].

Although, in *C*. *elegans*, there is no evidence of mitochondria outer membrane permeabilization by PTP and cytochrome *c* liberation during apoptosis, the mitochondrial network does seem affected and tubular mitochondria become fissioned [62]. There is also evidence that factors like WAH-1 [63] and CPS-6 [64] are released from the mitochondria and are important for cell apoptosis execution [65]. This structural change enables the hypothesis that high Δ*ψ*_m_ might conserve an orthodox configuration that decreases apoptotic events [66]. Our data suggest that Δ*ψ*_m_ might play a less important role during somatic apoptosis in *C*. *elegans*, but it is important to protect germ cells from this type of cell death.

### MAI-2 protects *C*. *elegans* from stress

In our work, we studied the impact on the survival of whole organisms deficient in IF_1_ (MAI-2) when threatened with cyanide (a mitochondrial complex IV inhibitor), CCCP (an uncoupler), heat stress and paraquat, a mitochondrial ROS generator. We found that *mai-2* mutants were more sensitive to cyanide or CCCP (Fig 8A and 8B), and less, but consistently, sensitive to heat shock or oxidative stress (Fig 8C and 8D). *C*. *elegans* is prone to encounter different stressors in the wild such as changes in temperature, unevenly distribution of oxygen in the soil or microbial pathogens such as *Pseudomona aeruginosa* that produce cyanide as a primary toxic factor that kills the nematode [44]. Due to these circumstances, we hypothesized that MAI-2 is necessary to protect whole animals from stress. Similarly, Fujikawa *et al*. and Campanella *et al*. observed that IF_1_-KD cells died at a higher rate than wild-type under fatal conditions such as paraquat and cyanide [4,17]. By contrast, Chen *et al*. [14] showed that, in the absence of IF_1_, cells showed increased survival when treated with antimycin, a complex III inhibitor.

Our work showed that MAI-2 plays an important role in apoptosis, probably by regulating Δ*ψ*_m_. Additionally, we demonstrated that IF_1_ function is mainly observed under stress conditions and that, under physiological conditions, this protein apparently does not play an essential role. Our work urges the relevance of studying the role of IF_1_ under stress conditions in organism models like zebrafish and mice in which mutants are available. Although we focused on the study of MAI-2, due to the lack of an MAI-1 mutant, it will be interesting to study the function of MAI-1 because its expression is unusual.

## Supporting information

S1 Fig MAI-1::mCherry localizes in the hypodermis, rectum, vulva, neurons and coelomocytes. One-day-old adult animals expressing the transgene *Pmai-1*::*mai-1*::*mCherry-mai-1 3’UTR* were observed under a confocal microscope. We observed the expression of mCherry::MAI-1 in the cytoplasm and nuclei of the hypodermis (hyp, shown with arrows) (A-B), in the rectum (rect), vulva, coelomocytes (cc) and neurons (C-D).
(TIF)

S2 Fig F_1_F_o_ATPase activity of the monomeric and dimeric F_1_F_o_ ATPase complex in *C*. *elegans* mitochondria. F_1_F_o_ATPase purified extracts from a mix population of L4/young adult animals of the indicated background were loaded into blue native gels and ATPase activity was assessed after 2 h of incubation. Bt = *Bos taurus* F_1_F_o_ATPase was used as loading control.
(TIF)

S1 Table Primers used for the construction of transgenes and CRISPR-Cas-9 genome editing. (PDF)

## References

[1] Mitchell P, Moyle J. Chemiosmotic hypothesis of oxidative phosphorylation. Nature. 1967;213: 137–139. doi: 10.1038/213137a0 4291593 10.1038/213137a0

[2] Boyer PD. The ATP synthase—a splendid molecular machine. Annu Rev Biochem. 1997;66: 717–49. doi: 10.1146/annurev.biochem.66.1.717 9242922 10.1146/annurev.biochem.66.1.717

[3] Pullman ME, Monroy GC. a Naturally Occurring Inhibitor of Mitochondrial Adenosine Triphosphatase. J Biol Chem. 1963;238: 3762–9. Available: http://www.ncbi.nlm.nih.gov/pubmed/14109217 14109217

[4] Campanella M, Casswell E, Chong S, Farah Z, Wieckowski MR, Abramov AY, et al Regulation of Mitochondrial Structure and Function by the F1Fo-ATPase Inhibitor Protein, IF1. Cell Metab. 2008;8: 13–25. doi: 10.1016/j.cmet.2008.06.001 18590689 10.1016/j.cmet.2008.06.001

[5] Lefebvre V, Du Q, Baird S, Ng ACH, Nascimento M, Campanella M, et al Genome-wide RNAi screen identifies ATPase inhibitory factor 1 (ATPIF1) as essential for PARK2 recruitment and mitophagy. Autophagy. 2013;9: 1770–1779. doi: 10.4161/auto.25413 24005319 10.4161/auto.25413

[6] Faccenda D, Campanella M. Molecular Regulation of the Mitochondrial F1Fo-ATPsynthase: Physiological and Pathological Significance of the Inhibitory Factor 1 (IF1). Int J Cell Biol. 2012;2012: 1–12. doi: 10.1155/2012/367934 22966230 10.1155/2012/367934 PMC3433140

[7] García-Bermúdez J, Cuezva JM. The ATPase Inhibitory Factor 1 (IF1): A master regulator of energy metabolism and of cell survival. Biochim Biophys Acta. 2016; doi: 10.1016/j.bbabio.2016.02.004 26876430 10.1016/j.bbabio.2016.02.004

[8] Campanella M, Seraphim A, Abeti R, Casswell E, Echave P, Duchen MR. IF1, the endogenous regulator of the F1Fo-ATPsynthase, defines mitochondrial volume fraction in HeLa cells by regulating autophagy. Biochim Biophys Acta—Bioenerg. 2009;1787: 393–401. doi: 10.1016/j.bbabio.2009.02.023 19269273 10.1016/j.bbabio.2009.02.023

[9] Garcia JJ, Morales-Rios E, Cortes-Hernandez P, Rodriguez-Zavala JS. The inhibitor protein (IF1) promotes dimerization of the mitochondrial F1F0-ATP synthase. Biochemistry. 2006;45: 12695–12703. doi: 10.1021/bi060339j 17042487 10.1021/bi060339j

[10] Buzhynskyy N, Sens P, Prima V, Sturgis JN, Scheuring S. Rows of ATP synthase dimers in native mitochondrial inner membranes. Biophys J. 2007;93: 2870–6. doi: 10.1529/biophysj.107.109728 17557793 10.1529/biophysj.107.109728 PMC1989723

[11] Faccenda D, Tan CH, Seraphim a, Duchen MR, Campanella M. IF1 limits the apoptotic-signalling cascade by preventing mitochondrial remodelling. Cell Death Differ. 2013;20: 686–97. doi: 10.1038/cdd.2012.163 23348567 10.1038/cdd.2012.163 PMC3619234

[12] Fujikawa M, Imamura H, Nakamura J, Yoshida M. Assessing actual contribution of IF1, inhibitor of mitochondrial FoF1, to ATP homeostasis, cell growth, mitochondrial morphology, and cell viability. J Biol Chem. 2012;287: 18781–7. doi: 10.1074/jbc.M112.345793 22493494 10.1074/jbc.M112.345793 PMC3365700

[13] Shah DI, Takahashi-Makise N, Cooney JD, Li L, Schultz IJ, Pierce EL, et al Mitochondrial Atpif1 regulates haem synthesis in developing erythroblasts. Nature Publishing Group; 2012;491: 608–12. doi: 10.1038/nature11536 23135403 10.1038/nature11536 PMC3504625

[14] Chen WW, Birsoy K, Mihaylova MM, Snitkin H, Stasinski I, Yucel B, et al Inhibition of ATPIF1 ameliorates severe mitochondrial respiratory chain dysfunction in mammalian cells. Cell Rep. 2014;7: 27–34. doi: 10.1016/j.celrep.2014.02.046 24685140 10.1016/j.celrep.2014.02.046 PMC4040975

[15] Barbato S, Sgarbi G, Gorini G, Baracca A, Solaini G. The Inhibitor Protein (IF1) of the F1F0-ATPase Modulates Human Osteosarcoma Cell Bioenergetics. J Biol Chem. 2015;290: 6338–48. doi: 10.1074/jbc.M114.631788 25605724 10.1074/jbc.M114.631788 PMC4358270

[16] Faccenda D, Nakamura J, Gorini G, Dhoot GK, Faccenda D, Nakamura J, et al Control of Mitochondrial Remodeling by the ATPase Inhibitory Factor 1 Unveils a Pro-survival Relay Report Control of Mitochondrial Remodeling by the ATPase Inhibitory Factor 1 Unveils a Pro-survival Relay via OPA1. CellReports. ElsevierCompany.; 2017;18: 1869–1883. doi: 10.1016/j.celrep.2017.01.070 28228254 10.1016/j.celrep.2017.01.070

[17] Nakamura J, Fujikawa M, Yoshida M. IF1, a natural inhibitor of mitochondrial ATP synthase, is not essential for the normal growth and breeding of mice. Biosci Rep. 2013;33: 10–12. doi: 10.1042/BSR20130078 23889209 10.1042/BSR20130078 PMC3775512

[18] Spieth J, Brooke G, Kuersten S, Lea K, Blumenthal T. Operons in *C*. *elegans*: Polycistronic mRNA precursors are processed by trans-splicing of SL2 to downstream coding regions. Cell. 1993;73: 521–532. doi: 10.1016/0092-8674(93)90139-H 8098272 10.1016/0092-8674(93)90139-h

[19] Ichikawa N, Ando C, Fumino M. *Caenorhabditis elegans* MAI-1 protein, which is similar to mitochondrial ATPase inhibitor (IF1), can inhibit yeast F0F1-ATPase but cannot be transported to yeast mitochondria. J Bioenerg Biomembr. 2006;38: 93–9. doi: 10.1007/s10863-006-9009-2 16897438 10.1007/s10863-006-9009-2

[20] Brenner S. The genetics of *Caenorhabditis elegans*. Genetics. 1974;77: 71–94. 4366476 10.1093/genetics/77.1.71 PMC1213120

[21] Sun AY, Lambie EJ. gon-2, a gene required for gonadogenesis in *Caenorhabditis elegans*. Genetics. 1997;147: 1077–1089. 9383054 10.1093/genetics/147.3.1077 PMC1208235

[22] Heckman KL, Pease LR. Gene splicing and mutagenesis by PCR-driven overlap extension. Nat Protoc. 2007;2: 924–932. doi: 10.1038/nprot.2007.132 17446874 10.1038/nprot.2007.132

[23] Maduro M, Pilgrim D. Identification and cloning of unc-119, a gene expressed in the *Caenorhabditis elegans* nervous system. Genetics. 1995;141: 977–988. 8582641 10.1093/genetics/141.3.977 PMC1206859

[24] Praitis V, Casey E, Collar D, Austin J. Creation of low-copy integrated transgenic lines in *Caenorhabditis elegans*. Genetics. 2001;157: 1217–1226. 11238406 10.1093/genetics/157.3.1217 PMC1461581

[25] Paz-Gómez D, Villanueva-Chimal E, Navarro RE. The DEAD Box RNA helicase VBH-1 is a new player in the stress response in *C*. *elegans*. PLoS One. 2014;9: e97924 doi: 10.1371/journal.pone.0097924 24844228 10.1371/journal.pone.0097924 PMC4028217

[26] Frøkjaer-Jensen C, Davis MW, Hopkins CE, Newman BJ, Thummel JM, Olesen S-P, et al Single-copy insertion of transgenes in *Caenorhabditis elegans*. Nat Genet. 2008;40: 1375–1383. doi: 10.1038/ng.248 18953339 10.1038/ng.248 PMC2749959

[27] Frøkjær-Jensen C, Davis MW, Ailion M, Jorgensen EM. Improved Mos1-mediated transgenesis in *C*. *elegans*. Nat Methods. 2012;9: 117–118. doi: 10.1038/nmeth.1865 22290181 10.1038/nmeth.1865 PMC3725292

[28] Schneider C a, Rasband WS, Eliceiri KW. NIH Image to ImageJ: 25 years of image analysis. Nat Methods. 2012;9: 671–675. doi: 10.1038/nmeth.2089 22930834 10.1038/nmeth.2089 PMC5554542

[29] Friedland AE, Tzur YB, Esvelt KM, Colaiácovo MP, Church GM, Calarco J a. Heritable genome editing in *C*. *elegans* via a CRISPR-Cas9 system. Nat Methods. 2013;10: 741–3. doi: 10.1038/nmeth.2532 23817069 10.1038/nmeth.2532 PMC3822328

[30] Paix A, Wang Y, Smith HE, Lee C-YS, Calidas D, Lu T, et al Scalable and Versatile Genome Editing Using Linear DNAs with Micro-Homology to Cas9 Sites in *Caenorhabditis elegans*. Genetics. 2014;198: 1347–1356. doi: 10.1534/genetics.114.170423 25249454 10.1534/genetics.114.170423 PMC4256755

[31] Yee C, Yang W, Hekimi S. The intrinsic apoptosis pathway mediates the pro-longevity response to mitochondrial ROS in *C elegans*. Cell. 2014;157: 897–909. doi: 10.1016/j.cell.2014.02.055 24813612 10.1016/j.cell.2014.02.055 PMC4454526

[32] Carlos Giovanni SG, Rosa EN. The *C*. *elegans* TIA-1/TIAR homolog TIAR-1 is required to induce germ cell apoptosis. Genesis. 2013;51: 690–707. doi: 10.1002/dvg.22418 23913578 10.1002/dvg.22418

[33] Yang W, Hekimi S. A Mitochondrial Superoxide Signal Triggers Increased Longevity in *Caenorhabditis elegans*. PLoS Biol. 2010;8: e1000556 doi: 10.1371/journal.pbio.1000556 21151885 10.1371/journal.pbio.1000556 PMC2998438

[34] Rolland SG. How to Analyze Mitochondrial Morphology in Healthy Cells and Apoptotic Cells in *Caenorhabditis elegans*. 2014;544: 75–98. doi: 10.1016/B978-0-12-417158-9.00004-2 10.1016/B978-0-12-417158-9.00004-2 24974287

[35] Palikaras K, Lionaki E, Tavernarakis N. Coordination of mitophagy and mitochondrial biogenesis during ageing in *C*. *elegans*. Nature. 2015;521: 525–528. doi: 10.1038/nature14300 25896323 10.1038/nature14300

[36] Grad LI, Sayles LC, Lemire BD. Isolation and functional analysis of mitochondria from the nematode *Caenorhabditis elegans*. Methods Mol Biol. 2007;372: 51–66. doi: 10.1007/978-1-59745-365-3_4 18314717 10.1007/978-1-59745-365-3_4

[37] Bollag DM, Edelstein SJ. Protein methods. 2Ed, Wiley-Liss, Inc, New York, 1996.

[38] Markwell MA, Haas SM, Bieber LL, Tolbert NE. A modification of the Lowry procedure to simplify protein determination in membrane and lipoprotein samples. Anal Biochem. 1978;87: 206–210. doi: 10.1016/0003-2697(78)90586-9 98070 10.1016/0003-2697(78)90586-9

[39] Schägger H, von Jagow G. Blue native electrophoresis for isolation of membrane protein complexes in enzymatically active form. Anal Biochem. 1991;199: 223–231. doi: 10.1016/0003-2697(91)90094-A 1812789 10.1016/0003-2697(91)90094-a

[40] Schägger H, von Jagow G. Tricine-sodium dodecyl sulfate-polyacrylamide gel electrophoresis for the separation of proteins in the range from 1 to 100 kDa. Anal Biochem. 1987;166: 368–379. doi: 10.1016/0003-2697(87)90587-2 2449095 10.1016/0003-2697(87)90587-2

[41] Yoshida M, Sone N, Hirata H, Kagawa Y. A highly stable adenosine triphosphatase from a thermophillie bacterium. Purification, properties, and reconstitution. J Biol Chem. 1975;250: 7910–6. Available: http://www.ncbi.nlm.nih.gov/pubmed/240842 240842

[42] Salinas LS, Maldonado E, Navarro RE. Stress-induced germ cell apoptosis by a p53 independent pathway in *Caenorhabditis elegans*. C1 Salinas LS, Maldonado E, Navarro RE Stress germ cell apoptosis by a p53 Indep Pathw *Caenorhabditis elegans* Cell Death Differ,2006;13(12)2129–39 doi: 10.1038/sj.cdd.4401976 16729024 10.1038/sj.cdd.4401976

[43] Arun P, Moffett JR, Ives JA, Todorov TI, Centeno JA, Namboodiri MAA, et al Rapid sodium cyanide depletion in cell culture media: Outgassing of hydrogen cyanide at physiological pH. Anal Biochem. 2005;339: 282–289. doi: 10.1016/j.ab.2005.01.015 15797569 10.1016/j.ab.2005.01.015

[44] Saldanha JN, Parashar A, Pandey S, Powell-Coffman JA. Multiparameter behavioral analyses provide insights to mechanisms of cyanide resistance in *Caenorhabditis elegans*. Toxicol Sci. 2013;135: 156–168. doi: 10.1093/toxsci/kft138 23805000 10.1093/toxsci/kft138 PMC3748764

[45] Labbadia J, Morimoto RI. Repression of the Heat Shock Response Is a Programmed Event at the Onset of Reproduction. Mol Cell. Elsevier Inc.; 2015;59: 1–12. doi: 10.1016/j.molcel.2015.06.027 26212459 10.1016/j.molcel.2015.06.027 PMC4546525

[46] Wilkinson DS, Taylor RC, Dillin A. Analysis of Aging in *Caenorhabditis elegans*. Methods Cell Biol. 2012;107: 353–381. doi: 10.1016/B978-0-12-394620-1.00012-6 22226530 10.1016/B978-0-12-394620-1.00012-6

[47] Merritt C, Rasoloson D, Ko D, Seydoux G. Report 3 0 UTRs Are the Primary Regulators of Gene Expression in the *C*. *elegans* Germline. 2008; 1476–1482. 10.1016/j.cub.2008.08.013 PMC2585380 18818082

[48] Pulak R, Anderson P. mRNA surveillance by the *Caenorhabditis elegans smg* genes. Genes Dev. 1993;7: 1885–1897. doi: 10.1101/gad.7.10.1885 8104846 10.1101/gad.7.10.1885

[49] Andreux PA, Mouchiroud L, Wang X, Jovaisaite V, Mottis A, Bichet S, et al A method to identify and validate mitochondrial modulators using mammalian cells and the worm *C*. *elegans*. Sci Rep. 2014;4: 5285 doi: 10.1038/srep05285 24923838 10.1038/srep05285 PMC4055904

[50] Braeckman BP, Houthoofd K, De Vreese A, Vanfleteren JR. Assaying metabolic activity in ageing *Caenorhabditis elegans*. Mech Ageing Dev. 2002;123: 105–119. doi: 10.1016/S0047-6374(01)00331-1 11718805 10.1016/s0047-6374(01)00331-1

[51] Liu X, Long F, Peng H, Aerni SJ, Jiang M, Sánchez-Blanco A, et al Analysis of Cell Fate from Single-Cell Gene Expression Profiles in *C*. *elegans*. Cell. 2009;139: 623–633. doi: 10.1016/j.cell.2009.08.044 19879847 10.1016/j.cell.2009.08.044 PMC4709123

[52] Schumacher B, Schertel C, Wittenburg N, Tuck S, Mitani S, Gartner a, et al *C*. *elegans* ced-13 can promote apoptosis and is induced in response to DNA damage. Cell Death Differ. 2005;12: 153–61. doi: 10.1038/sj.cdd.4401539 15605074 10.1038/sj.cdd.4401539

[53] Twig G, Elorza A, Molina AJA, Mohamed H, Wikstrom JD, Walzer G, et al Fission and selective fusion govern mitochondrial segregation and elimination by autophagy. EMBO J. 2008;27: 433–446. doi: 10.1038/sj.emboj.7601963 18200046 10.1038/sj.emboj.7601963 PMC2234339

[54] Schleyer M, Schmidt B, Neupert W. Requirement of a Membrane Potential for the Posttranslational Transfer of Proteins into Mitochondsria. Eur J Biochem. 1982;125: 109–116. doi: 10.1111/j.1432-1033.1982.tb06657.x 6213410 10.1111/j.1432-1033.1982.tb06657.x

[55] Ly JD, Grubb DR, Lawen a. The mitochondrial membrane potential (deltapsi(m)) in apoptosis; an update. Apoptosis. 2003;8: 115–128. 12766472 10.1023/a:1022945107762

[56] Tait SWG, Green DR. Mitochondria and cell death: outer membrane permeabilization and beyond. Nat Publ Gr. Nature Publishing Group; 2010;11: 621–632. doi: 10.1038/nrm2952 20683470 10.1038/nrm2952

[57] Petronilli V, Cola C, Massari S, Colonna R, Bernardi P. Physiological effectors modify voltage sensing by the cyclosporin A- sensitive permeability transition pore of mitochondria. J Biol Chem. 1993;268: 21939–21945. 8408050

[58] Schertel C, Conradt B. C. elegans orthologs of components of the RB tumor suppressor complex have distinct pro-apoptotic functions. Development. 2007;134: 3691–3701. doi: 10.1242/dev.004606 17881492 10.1242/dev.004606

[59] Gumienny TL, Lambie E, Hartwieg E, Horvitz HR, Hengartner MO. Genetic control of programmed cell death in the *Caenorhabditis elegans* hermaphrodite germline. Development. 1999;126: 1011–1022. 9927601 10.1242/dev.126.5.1011

[60] Schumacher B, Hofmann K, Boulton S, Gartner A. The *C*. *elegans* homolog of the p53 tumor suppressor is required for DNA damage-induced apoptosis. Curr Biol. 2001;11: 1722–1727. 11696333 10.1016/s0960-9822(01)00534-6

[61] Láscarez-Lagunas LI, Silva-García CG, Dinkova TD, Navarro RE. LIN-35/Rb causes starvation-induced germ cell apoptosis via CED-9/Bcl2 downregulation in C. elegans. Mol Cell Biol. 2014;34: 2499–2516. doi: 10.1128/MCB.01532-13 24752899 10.1128/MCB.01532-13 PMC4054308

[62] Jagasia R, Grote P, Westermann B, Conradt B. DRP-1-mediated mitochondrial fragmentation during EGL-1-induced cell death in *C*. *elegans*. Nature 2005;433(7027):754–60. doi: 10.1038/nature03316 15716954 10.1038/nature03316

[63] Wang X, Yang C, Chai J, Shi Y. Mechanisms of AIF-Mediated Apoptotic DNA Degradation in Caenorhabditis elegans. Science. 2002;298: 1587–1592. doi: 10.1126/science.1076194 12446902 10.1126/science.1076194

[64] Parrish J, Li L, Klotz K, Ledwich D, Wang X, Xue D. Mitochondrial endonuclease G is important for apoptosis in *C*. *elegans*. Nature. 2001;412: 0–4. 10.1038/35083608 11452313

[65] Seervi M, Xue D. Mitochondrial Cell Death Pathways in *Caenorhabiditis elegans* [Internet]. 1st ed Apoptosis and Development. Elsevier Inc; 2015 doi: 10.1016/bs.ctdb.2015.07.019 10.1016/bs.ctdb.2015.07.019 26431563

[66] Gottlieb E, Armour S, Harris M, Thompson C. Mitochondrial membrane potential regulates matrix configuration and cytochrome c release during apoptosis. Cell Death Differ. 2003;10: 709–717. doi: 10.1038/sj.cdd.4401231 12761579 10.1038/sj.cdd.4401231

