# Supplementary figures and images for: Caenorhabditis elegans ATPase inhibitor factor 1 (IF1) MAI-2 preserves the mitochondrial membrane potential (Δψm) and is important to induce germ cell apoptosis
Source: PLoS One. 2017 Aug 22;12(8):e0181984. doi: 10.1371/journal.pone.0181984 (PMC5568743; doi:10.1371/journal.pone.0181984)

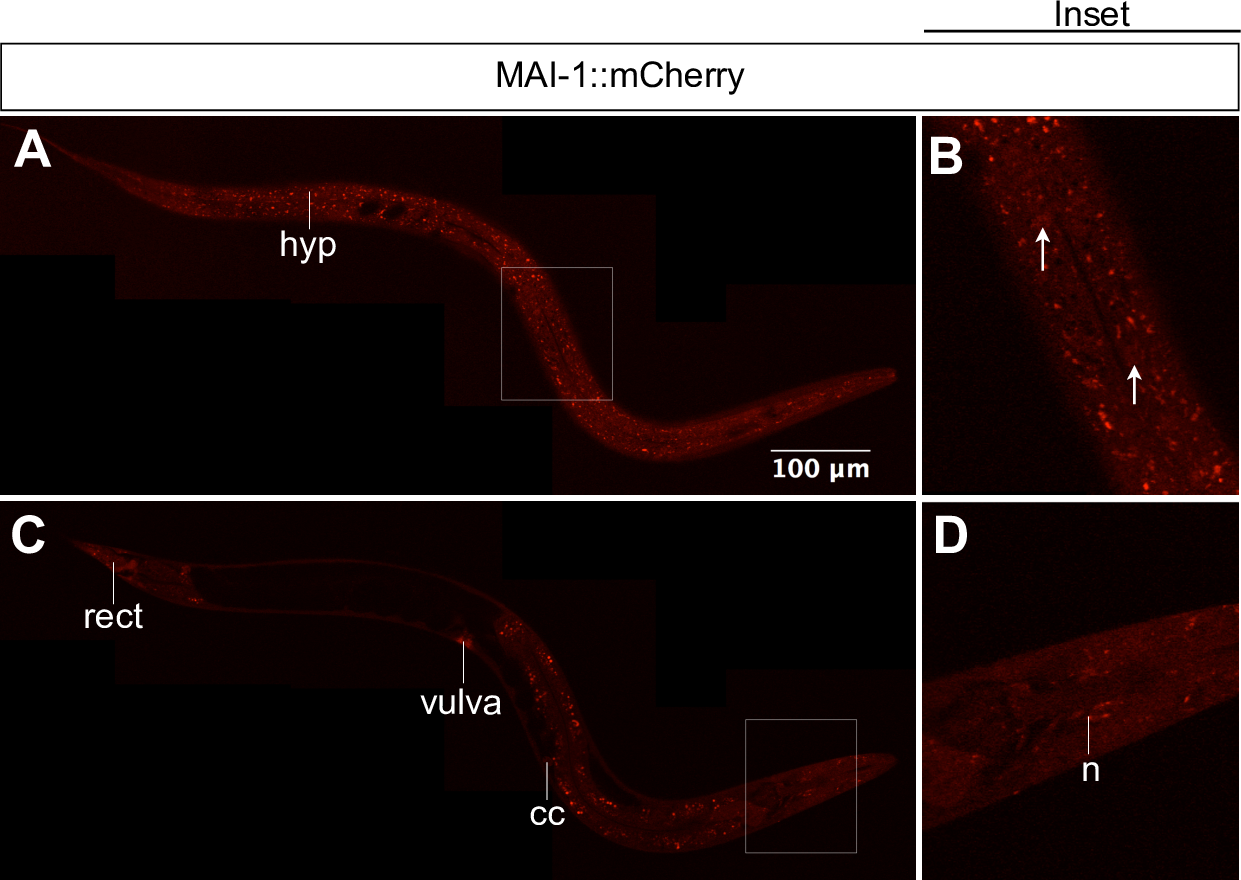

Supplement: S1 Fig — One-day-old adult animals expressing the transgene Pmai-1::mai-1::mCherry-mai-1 3’UTR were observed under a confocal microscope. We observed the expression of mCherry::MAI-1 in the cytoplasm and nuclei of the hypodermis (hyp, shown with arrows) (A-B), in the rectum (rect), vulva, coelomocytes (cc) and neurons (C-D). (TIF) [file pone.0181984.s001.tif]

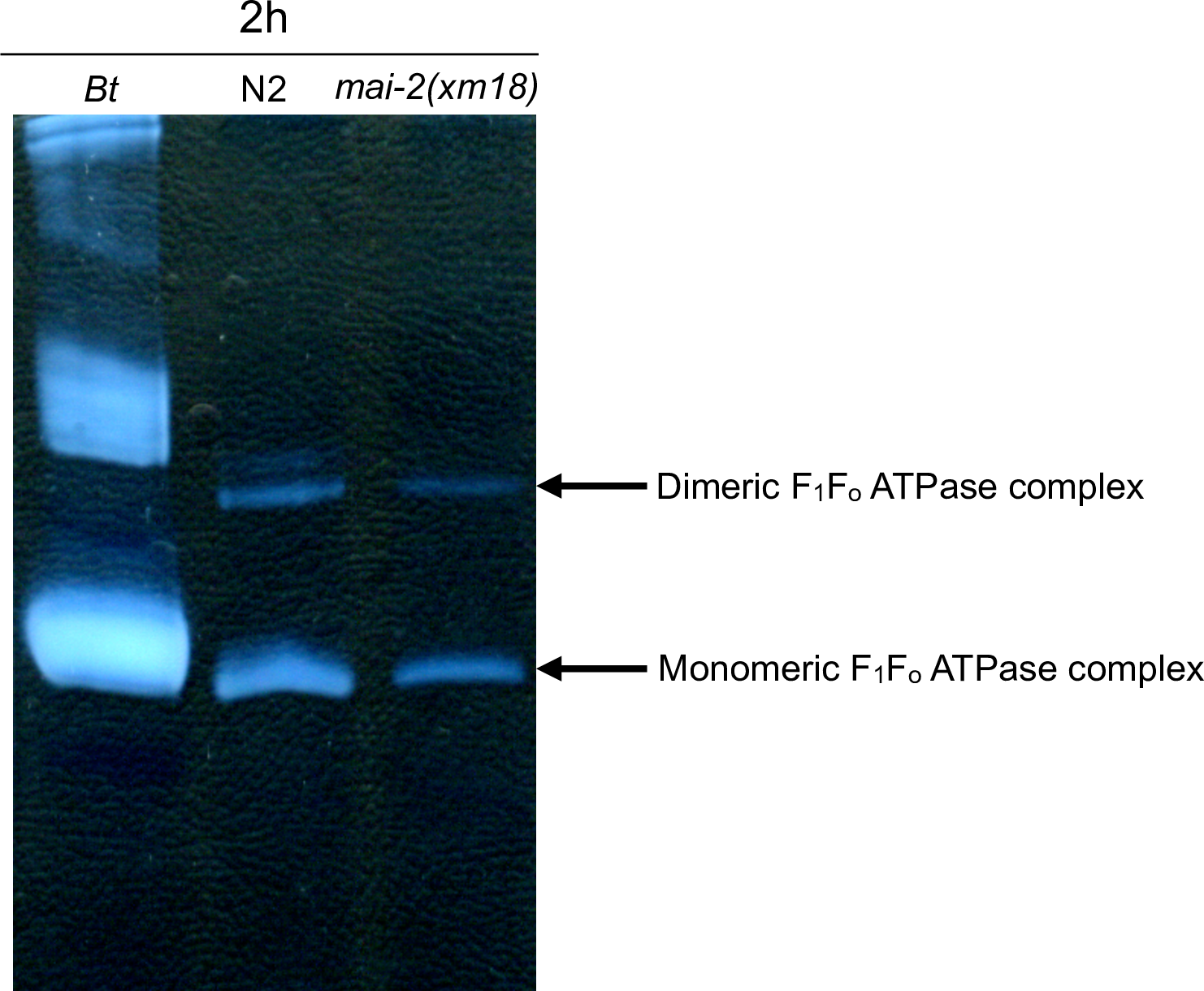

Supplement: S2 Fig — F1FoATPase purified extracts from a mix population of L4/young adult animals of the indicated background were loaded into blue native gels and ATPase activity was assessed after 2 h of incubation. Bt = Bos taurus F1FoATPase was used as loading control. (TIF) [file pone.0181984.s002.tif]
