# Supplementary material for: Caenorhabditis elegans ATPase inhibitor factor 1 (IF1) MAI-2 preserves the mitochondrial membrane potential (Δψm) and is important to induce germ cell apoptosis
Source: PLoS One. 2017 Aug 22;12(8):e0181984. doi: 10.1371/journal.pone.0181984 (PMC5568743; doi:10.1371/journal.pone.0181984)
Supplement: S1 Table — (PDF) [file pone.0181984.s003.pdf]

S1 Table. Primers used for the construction of transgenes and CRISPR-Cas-9 genome editing.

Use	Primer	Sequence
Pmai-2::mai-2::GFP::mai-2 3'UTR	*Pmai-2* F	5'-GGGGACAACTTTGTATAGAAAAGTTGATTTCGTTTGGTAGACGAGG-3'
	Pmai-2 R	5'-GGGGACTGCTTTTTTGTACAAACTTGATTCTGAAAATTGAGTGAATTAGAGAG-3'
	mai-2 F	5'-GGGGACAAGTTTGTACAAAAAAGCAGGCTCAATGCTCAGCGTTTCAAGAGCCG-3'
	mai-2 R	5'-GGGGACCACTTTGTACAAGAAAGCTGGGTTTCCTTCCTTTCCGAGGGCACGC-3'
	gfp F	5'-GGGGACAGCTTTCTTGTACAAAGTGGGAATGAGTAAAGGAGAAGAACTTTTCACTG-3'
	gfp R	5'-GACTAGCTCGCGTTCTTGTACTGCAAATTATTTGTATAGTTCGTCCATGCCATG-3'
	*mai-2 3′*UTR F	5'-CATGGCATGGACGAACTATACAAATAA TTTGCAGTACAAGAACGCGAGCTAGTC-3'
	*mai-2 3′*UTR R	5'-GGGGACAACTTTGTATAATAAAGTTG CCTCTATAATTTTCCTCTATAAAGCTTGTC-3'
Pmai-1::GFP::mai-1::mai-1 3'UTR	*Pmai-1* F	5'-GGGGACAACTTTGTATAGAAAAGTTGTTTGTTTTGATGTGCTTT-3'
	Pmai-1 R	5'-GGGGACTGCTTTTTTGTACAAACTTGTCATCTGTTTTGATTTCCTG-3'
	mai-1 F	5'-GGGGACAAGTTTGTACAAAAAAGCAGGCTCAATGTCAGGATCTGGAAGTG-3'
	mai-1 R	5'-GGGGACAACTTTGTATAATAAAGTTGGGACAAGACGTCCGATTC-3'
Pmai-1::mai-1::mCherry::mai-1 3'UTR	*Pmai-1* F	5'-GGGGACAACTTTGTATAGAAAAGTTG TTTGTTTTGATGTGCTTT-3'
	Pmai-1 R	5'-GGGGACTGCTTTTTTGTACAAACTTGTCATCTGTTTTGATTTCCTG-3'
	mai-1 F	5'-GGGGACAAGTTTGTACAAAAAAGCAGGCTCAATGTCAGGATCTGGAAGTG-3'
	mai-1 R	5'-GGGGACCACTTTGTACAAGAAAGCTGGGTTTCCTGTTCCGTGTCCTTTCTCGA-3'
	mCherry F	5'-GGGGACAGCTTTCTTGTACAAAGTGGGAATGGTCTCAAAGGGTGAAGAA-3'
	mCherry R	5' AGAGCCGTTTGAAAAGCCCTCTATTTTTTACTTATACAATTCATCCATGCCACC-3'
	mai-1-3'UTR F	5'-GGTGGCATGGATGAATTGTATAAGTAAAAAATAGAGGGCTTTTCAAACGGCTCT-3'
	mai-1-3'UTR R	5'-GGGGACAAC TTTGTATAATAAAGTTGGGACAAGACGTCCGATTC-3'
Pmex-5::tomm-20::mcherry::tbb-2 3'UTR	*tomm-20* F	5'-GGGGACAAGTTTGTACAAAAAAGCAGGCTTAAAAATGTCGGACACAATTCTTG-3'
	tomm-20 R	5'-TTCTTCACCCTTTGAGACCATTCCAGCCTGGGCACG-3'
	mCherry F	5'-AGAGACGTGCCCAGGCTGGAATGGTCTCAAAGGGTGAAGAAG-3'
	mCherry R	5'-GGGGACCACTTTGTACAAGAAAGCTGGGTACTTATACAATTCATCCATGCCACC-3'.
mai-2 editing by CRISPR/Cas9	*mai-2 sgRNA* F	5'-GGATCGATCCGCGACGCCGGGTTTTAGAGCTAGAAATAGCAAGTTAAAATAAG-3
	mai-2 sgRNA R	5'-CCCGGCGTCGCGGATCGATCCAAACATTTAGATTTGCAATTCAATTATATAGG-3'.
	mai-2 ssDNA1	5'GGAATGGTCGCCCGCTTCTCCGCCGGAGGACACGGAGACGGAGCCGGACGCGGAGGA GGCTCCGGTGGATCGATCCGCGACGCCGGCAGCTGAGGTGCGTTCGGAAAGATGGAGGC CGCCCGCGAGGACGAGTACTTCTACAAGAAGCAGAAGGCTCAACT-3'
	mai-2 ssDNA2	5'CGGAATGGTCGCCCGCTTCTCCGCCGGAGGACACGGAGACGGAGCCGGACGCGGAGG AGGCTCCGGTTAATAATTTGCAGTACAAGAACGCGAGCTAGTCGTCTTCTGAAATTTTCTT CATTTTTTC-3'
mai-2(xm18) and *mai-2(xm19)* detection	*mai-2* F	5′-TTTCGCAAGTTTCAGGCAG-3'
	*mai-2(xm*18) R	5'-ATTGTTCAGCTCCCGCAC-3'
	*mai-2(xm19)*R	5'-GGTCTCGAAGTGGAAGAAA-3'
RTPCR	*mai-2* F	5'-GCTCTAGAATGCTCAGCGTTTCAAGA-3'
	mai-2(xm18) R	5'-GCGGATCCTTATTCCTTTCCGAGGGC-3'
	mai-2(xm19) R	5'-CACGTGGCGAAAATAGAGAGAG-3'
